# Bayesian hierarchical piecewise regression models: a tool to detect trajectory divergence between groups in long-term observational studies

**DOI:** 10.1186/s12874-017-0358-9

**Published:** 2017-06-06

**Authors:** Marie-jeanne Buscot, Simon S. Wotherspoon, Costan G. Magnussen, Markus Juonala, Matthew A. Sabin, David P. Burgner, Terho Lehtimäki, Jorma S. A. Viikari, Nina Hutri-Kähönen, Olli T. Raitakari, Russell J. Thomson

**Affiliations:** 1 Menzies Institute for Medical Research, University of Tasmania, Hobart, Australia; 2 Institute of Marine and Antarctic Studies, University of Tasmania, Hobart, Australia; 3 Research Centre of Applied and Preventive Cardiovascular Medicine, University of Turku, Turku, Finland; 4 Department of Medicine, University of Turku and Division of Medicine, Turku University Hospital, Turku, Finland; 5 Murdoch Childrens Research Institute, The Royal Children’s Hospital, Melbourne, Australia; 6 Department of Paediatrics, University of Melbourne, Melbourne, Australia; 7 Department of Paediatrics, Monash Medical Centre, Melbourne, Australia; 8 Department of Clinical Chemistry, Fimlab Ltd and University of Tampere School of Medicine, Tampere, Finland; 9 Department of Pediatrics, University of Tampere School of Medicine, Tampere, Finland; 10 Tampere University Hospital, Tampere, Finland; 11 Centre for Research in Mathematics, School of Computing, Engineering & Mathematics, Western Sydney University, Sydney, Australia

**Keywords:** Piecewise model, Hierarchical regression, Non-linear trajectory model, Accelerated longitudinal design, Cohort effect, Group divergence

## Abstract

**Background:**

Bayesian hierarchical piecewise regression (BHPR) modeling has not been previously formulated to detect and characterise the mechanism of trajectory divergence between groups of participants that have longitudinal responses with distinct developmental phases. These models are useful when participants in a prospective cohort study are grouped according to a distal dichotomous health outcome. Indeed, a refined understanding of how deleterious risk factor profiles develop across the life-course may help inform early-life interventions. Previous techniques to determine between-group differences in risk factors at each age may result in biased estimate of the age at divergence.

**Methods:**

We demonstrate the use of Bayesian hierarchical piecewise regression (BHPR) to generate a point estimate and credible interval for the age at which trajectories diverge between groups for continuous outcome measures that exhibit non-linear within-person response profiles over time. We illustrate our approach by modeling the divergence in childhood-to-adulthood body mass index (BMI) trajectories between two groups of adults with/without type 2 diabetes mellitus (T2DM) in the Cardiovascular Risk in Young Finns Study (YFS).

**Results:**

Using the proposed BHPR approach, we estimated the BMI profiles of participants with T2DM diverged from healthy participants at age 16 years for males (95% credible interval (CI):13.5–18 years) and 21 years for females (95% CI: 19.5–23 years). These data suggest that a critical window for weight management intervention in preventing T2DM might exist before the age when BMI growth rate is naturally expected to decrease. Simulation showed that when using pairwise comparison of least-square means from categorical mixed models, smaller sample sizes tended to conclude a later age of divergence. In contrast, the point estimate of the divergence time is not biased by sample size when using the proposed BHPR method.

**Conclusions:**

BHPR is a powerful analytic tool to model long-term non-linear longitudinal outcomes, enabling the identification of the age at which risk factor trajectories diverge between groups of participants. The method is suitable for the analysis of unbalanced longitudinal data, with only a limited number of repeated measures per participants and where the time-related outcome is typically marked by transitional changes or by distinct phases of change over time.

**Electronic supplementary material:**

The online version of this article (doi:10.1186/s12874-017-0358-9) contains supplementary material, which is available to authorized users.

## Background

Child- to-adult trajectories of health markers are likely to have implications for the risk of chronic diseases in later life, such as obesity, type 2 diabetes mellitus (T2DM) and cardiovascular diseases; it is therefore important to understand their development throughout the life-course [1–4].

Long-running observational studies that follow the same subjects participants across the life-course are especially suited to studying adult onset disorders, such as cardiometabolic disease, since they allow characterizing the development of normal vs. pathological processes overtime. A goal of such studies is often to determine how a number of patient characteristics, modifiable risk factors profiles [1, 5], their interactions and normal aging may impact the onset and progression of disease over time [6–8] in order to identify time periods of divergence in these factors [9–11].

A key statistical issue in these studies is often to determine whether the risk factor levels vary over time between and within groups of participants, and whether different groups are changing in a similar or different fashion over time [12, 13]. Depending on the study, the stratification of participants into groups can relate to participants’ characteristics or exposure (i.e. smoking status), intervention arm (i.e. control vs. medication), or it could be a later health outcome (i.e. disease status in mid-adulthood). When participants are grouped according to a distal dichotomous health outcome, longitudinal data provide the foundation to understand pathways to deleterious risk factor profiles, which may help inform the timing of interventions [8, 14, 15].

When it is established that groups of interest start with similar initial outcome levels, but do not change similarly overtime, it is often of interest to determine the point in time or age at which they start diverging in their trajectories [16–20]. Being able to determine how and when the change manifests between groups of participants is important, since it can help pinpoint periods in the life course that are critical in the development of abnormal risk factor profiles [21]. However, there is little methodological guidance in the literature on statistical techniques to achieve this, and several studies have noted a lack of relevant methods to investigate trajectory divergence between groups [20–22].

A common attempt is to fit a mixed model with time (or age) treated as categorical variable (i.e. non time-ordered/ordinated [23]) to retrieve linear predictions at each age for each group of interest from this model (i.e. means of least squares predictions, aka LS-means [24–26]), and to test for a group difference in these predictions using a number of contrasts (i.e. post-hoc pairwise comparisons). In this case, the age at which the difference between-groups emerge is often the age at which a significant between-group difference materializes in the LS-means [23, 27, 28]. Several studies have used this approach to determine at “what times the groups means are different” (e.g. between-subject effect or post-hoc pairwise group comparison, if there are more than two groups) and/or ‘at what times the means differ’ within each group (within-subject effect testing) [27, 29, 30]. However, even when adjustments are applied for multiple tests [27, 31–33], many authors advise against the unrestricted use of multiple comparisons among marginal means due to well-documented multiple testing issues, especially the increase in false positive rate as the number of hypothesis tests increases [30, 34–37]. Mixed models that assume an unstructured mean response by treating age or time as categorical variables tend to be over parameterized and may be inefficient at detecting main effects [38]. Another crucial disadvantage of this approach, is that it only tests for the difference in means between groups at each time point and does not provide any information on subject-specific response evolution in time [13, 39], so that the age (or point in time) at which the group difference manifests is ultimately a question of sample size and statistical power.

In contrast, continuous time models such as individual-based trajectory modeling methods, including mixed effect [12], hierarchical [40], multilevel [41] and the closely related structural equation and Latent Growth Curve models [42], have become invaluable tools to understand the natural history of health outcome as well as risk factor/determinant trajectories [14, 43–45]. They have advantages over traditional approaches to repeated-measure data analysis; their main feature being that they allow summarizing each participant’s outcome trajectory with a few trajectory parameters [39, 46]. In addition, they permit the explicitly modeling of inter-individual differences in intra-individual change, permitting inference regarding the average response trajectory over time and how this evolution may vary with participant characteristics (i.e. participant-level predictors) [47–50].

Despite their flexibility, these models are not often used to analyse sparse long-term observational data since accelerated longitudinal designs [14, 22, 51] and non-linear response overtime [44, 52–54] both introduce significant complexity into the growth curve modeling approach [55–58]. Indeed, being able to represent non-linear patterns with a relatively small number of measurement occasions per participants (often <10 time points) and be specific about where between-participant heterogeneity appears in those patterns is a statistical challenge.

Many applications often relied on higher order time (or age) polynomials or latent basis coefficients [14, 20, 44, 59–61], which strengths and limitations have been described elsewhere [9, 46, 62–65]. In the context of our study the polynomial parameterisation of the growth model does not specifically yield an age or point in time when the growth pattern is changing within-and between-groups. Alternatively, piecewise models, also known as linear splines or broken stick models, can be used to break up a non-linear or curvilinear growth trajectory into several separate linear components [66]. They are particularly useful to compare growth rates in different periods over time if the functional form of the response is characterised by different phases of development, or if there is a shift in the outcome trajectory at some point in the event window (i.e. an acceleration or a deceleration in the response change rate from one point in time (or age)) [67–73]. Piecewise linear trajectory models have been used to model ‘multiphase’ developmental processes primarily with ‘fixed’ transition points in a variety of applications in the frequentist multilevel [40, 45, 69, 74, 75] and structural equation modeling framework [42, 76]. Bayesian applications of these processes are often referred to as ‘random change point model’ where the position of individual breakpoints is also estimated, allowing for between-person variability in the transition points [77–86].

Few studies have, however, investigated the inclusion of categorical covariates or grouping variables as level 2 predictors of the variability in the change point, and the random Bayesian change point model has not, to our knowledge, been formulated to test specifically for the existence of a ‘trajectory divergence’ between two (or more) known groups of participants that have longitudinal responses characterised by distinct developmental phases. In this paper we illustrate the use of Bayesian hierarchical piecewise regression modeling to detect trajectory divergence between groups of participants using longitudinal BMI data from the Cardiovascular Risk in Young Finns (YFS) Study, a well phenotyped prospective cohort with measures from multiple time-points. Previously published work on this data, based on categorical mixed modeling, suggested that BMI levels became statistically different between those who develop T2DM in adulthood and those who did not from the age of 15 years [87]. We re-analyse this data set to demonstrate how the Bayesian method can be used to (1) model the BMI profiles to better understand the natural history of the BMI trajectories in those who do and do not develop T2DM in adulthood while controlling for potential cohort effects, and (2) obtain a refined estimate and confidence interval of the age at which the two groups start diverging from one another, translating into significantly different BMI from a certain age onwards. In addition, we conduct a series of short simulations to illustrate the difference in the estimates of age at divergence when using the traditional approach (i.e. pairwise comparisons of marginal means from a categorical mixed model) vs. the proposed trajectory modeling approach.

## Methods

### Statistical model

#### No-covariate model

To accommodate the curvilinear developmental pattern in an individual continuous response over time while providing an adequate representation of its developmental theory, we consider a linear-linear piecewise regression model as the functional form of change in the trajectory model. The ‘change point’ (CP) represents the age (or time) at which the transition to a different growth rate occurs. We consider the following unconditional (no covariates) multilevel model:

Level 1 model:

1.1 $$ Repons{e}_{i j}={b}_{0 i}+{b}_{1 i} ag{e}_{i j}.\left(1-{u}_{C{P}_i}\left( ag{e}_{i j}\right)\right)+{b}_{2 i}\left( ag{e}_{i j}- C{P}_i\right).{u}_{C{P}_i}\left( ag{e}_{i j}\right)+{\varepsilon}_{i j} $$

Level 2 model:

1.2 $$ \begin{array}{c}\hfill {b}_{0 i}={\beta}_{00}+{v}_{0 i}\hfill \\ {}\hfill {b}_{1 i}={\beta}_{10}+{v}_{1 i}\hfill \\ {}\hfill {b}_{2 i}={\beta}_{20}+{v}_{2 i}\hfill \\ {}\hfill C{P}_i= CP+{v}_{CPi}\hfill \end{array} $$

1.3 $$ \left(\begin{array}{c}\hfill \begin{array}{c}\hfill \begin{array}{c}\hfill {v}_{0 i}\hfill \\ {}\hfill {v}_{1 i}\hfill \end{array}\hfill \\ {}\hfill {v}_{2 i}\hfill \end{array}\hfill \\ {}\hfill {v}_{CP i}\hfill \end{array}\right)\sim N\left[\left(\begin{array}{c}\hfill 0\hfill \\ {}\hfill 0\hfill \\ {}\hfill 0\hfill \\ {}\hfill 0\hfill \end{array}\right),\left(\begin{array}{cccc}\hfill {\sigma}_{v0}^2\hfill & \hfill \dots \hfill & \hfill \cdots \hfill & \hfill \dots \hfill \\ {}\hfill {\sigma}_{v01}\hfill & \hfill {\sigma}_{v1}^2\hfill & \hfill \dots \hfill & \hfill \dots \hfill \\ {}\hfill {\sigma}_{v02}\hfill & \hfill {\sigma}_{v12}\hfill & \hfill {\sigma}_{v2}^2\hfill & \hfill \dots \hfill \\ {}\hfill {\sigma}_{v0 CP}\hfill & \hfill {\sigma}_{v1 CP}\hfill & \hfill {\sigma}_{v2 CP}\hfill & \hfill {\sigma}_{CP}^2\hfill \end{array}\right)\right] $$

Where at age *j* for participant *i*, *Response*_*ij*_ is the repeated continuous outcome measures, and *age*_*ij*_ is the corresponding time related variables centered around its grand mean. ${u}_{C{ P}_i}\left( ag{e}_{i j}\right)$ is a unit heavyside step function where ${u}_{C{ P}_i}\left( ag{e}_{i j}\right)$ =1 if *age*_*ij*_ ≥ *CP*_*i*_ and ${u}_{C{ P}_i}\left( ag{e}_{i j}\right)=0$ if *age*_*ij*_ < *CP*_*i*_. The random trajectory parameters *b*_0*i*_, *b*_1*i*_ and *b*_2*i*_ correspond to the individual intercept, slope before and slope after the person-specific change point *CP*_*i*_, respectively. For each person *i*, *b*_0*i*_ controls the individual baseline level (or initial status) for the response and its interpretation depends on the centering of the age variable (e.g. if age is centered around 25 years, *b*_0*i*_ will be the expected participant-level response at 25 years of age given they are in the first phase of growth *b*_1*i*_). *b*_1*i*_, *b*_2*i*_ and *CP*_*i*_, are the expected linear increase per year of age in the first phase of growth, the expected linear rate of increase after the change point, and age at which the linear perturbation to the initial trend occurs, respectively. *ε*_*ij*_ is the level-1 residual (i.e. random within-person error for person *i* at age *j*) and is independent and normally distributed (i.e. *ε*_*ij*_ ~ *iid N*(0, *σ*_*e*_^2^)). *v*_0*i*_, *v*_1*i*_, *v*_2*i*_ and *v*_*CPi*_ are the level-2 random effects, multivariate normally distributed with zero mean and variances *σ*_*v*0_^2^, *σ*_*v*1_^2^, *σ*_*v*2_^2^ and *σ*_*CP*_^2^ respectively and full covariance matrix as shown in 1.3 . *β*_00_, *β*_10_, *β*_20_ and *CP* are the fixed effects (i.e. population average of each trajectory parameter). In this model, the level 1 residual variance *σ*_*e*_^2^ can be interpreted as the deviations around an individual’s trajectory and level-2 residuals as between-participant variability in the overall intercept (*σ*_*v*0_^2^), in the rate of change before and after the change point *CP*_*i*_ (*σ*_*v*1_^2^ and *σ*_*v*2_^2^ respectively), and in the change point itself (*σ*_*CP*_^2^), respectively.

#### Model with group-effect

To explore heterogeneity in individual trajectories between groups of interests, the unconditional segmented growth model can be expanded by including time-varying covariates (TVCs) at level-1 and time invariant covariates (TICs) at level 2, while simultaneously adjusting for the effects of variables measured on participants at all time points. Whereas TICs directly predict the growth parameters, TVCs directly predict the repeated measures while controlling for the influence of the growth parameters [43, 88]. If the TIC variable is a binary dummy grouping factor (“GRP_*i*_”), identifying participants coming from two identified groups, the model can be rewritten as follows:

Level 1 model:

2.1 $$ Respons{e}_{i j}={b}_{0 i}+{b}_{1 i} ag{e}_{i j}.\left(1-{u}_{C{P}_i}\left( ag{e}_{i j}\right)\right)+{b}_{2 i}\left( ag{e}_{i j}- C{P}_i\right).{u}_{C{P}_i}\left( ag{e}_{i j}\right)+ T V{C}_{i j}+{\varepsilon}_{i j} $$

Level 2 model:

2.2 $$ \begin{array}{c}\hfill {b}_{0 i}={\beta}_{00}+{\beta}_{0 grp} GR{P}_i+{v}_{0 i}\hfill \\ {}\hfill {b}_{1 i}={\beta}_{10}+{\beta}_{1 grp} GR{P}_i+{v}_{1 i}\hfill \\ {}\hfill {b}_{2 i}={\beta}_{20}+{\beta}_{2 grp} GR{P}_i+{v}_{2 i}\hfill \\ {}\hfill C{P}_i= CP+{\beta}_{CP} GR{P}_i+{v}_{CP i}\hfill \end{array} $$

Where *β*_00_, *β*_10_, *β*_20_ and CP are the expected trajectory parameters for the reference group (at zero values for other potential covariates); *β*_0*grp*_, *β*_1*grp*_, *β*_2*grp*_ *and β*_*CP*_ are the expected intergroup variations in these parameters for participants in the second group (i.e. respectively, in the mean response, in the linear age effect, in the deviation from linear rate after the CP and in the CP timing); and *v*_0*i*_, *v*_1*i*_, *v*_2*i*_ and *v*_*CPi*_ are the level-2 residuals person *i* for intercept, slopes, and age at the change point after controlling for group differences. To test for a between-group difference in one trajectory parameter only, ‘*GRP’* can be included as a level-2 predictor for the parameter of interest, and model all other growth parameters as random effects only (as in 1.2). For each of the *p* + 1 individual growth parameters, additional participant-specific covariates (TICs) can be included in a similar fashion to have multiple predictors at level 2 as follows: ${b}_{p i}={\beta}_{p0}+{\displaystyle \sum_{q=1}^{Qp}}{\beta}_{p q}{x}_{q i}+{u}_{p i}$, with *x*_*qi*_, the q^th^ measured TIC; *β*_*pq*_,the effect of the TIC *x*_*qi*_ on the *(p + 1)* ^*th*^ trajectory parameter; and *u*_*pi*_, the *(p + 1)* ^*th*^ random effect. The set of p + 1 random effects for person *i* assumed to be multivariate normally distributed with covariance matrix of dimension (*p* + 1) * (*p* + 1), although simpler variance-covariance structures of the random effects can be considered during model building (i.e. mutual independence of the random effects). It is advisory to standardize TVCs in order to stabilize the variance, improve normality of errors and linearity of the mean [88]. The common assumption for the error structure is *ε*_*ij*_ ~ *iid N*(0, *σ*_*e*_^2^) but it can be relaxed to include time specific variances or residual error correlation such as AR1 errors.

The same approach can be used to expand the hierarchical piecewise trajectory model with grouping factors that have more than 2 levels. This is one of the possible approaches to test for a cohort-effect on the development of curvilinear responses over time when data arises from multi-cohort or accelerated longitudinal designs [89, 90]. If study participants belong to one of *k* possible birth cohorts, *k-1* binary dummy variables are created to identify observations coming from people born in the same calendar year, and as in 2.2, these new *k-1* grouping variables are introduced as level 2 predictors of the different trajectory parameters in the model. The binary dummy variables are introduced to sequentially shift the conditional means of each of the different trajectory parameters. The fixed effects will be the average trajectory parameters for the cohort chosen as the reference cohort in the study sample, and each (*β*_*cohort*_)_1.. *k* − 1_ coefficient will thus be interpreted as the variation in growth parameters in the corresponding *k-1th* cohort compared to the reference cohort.

#### Trajectory divergence mechanisms

**Fig. 1:**
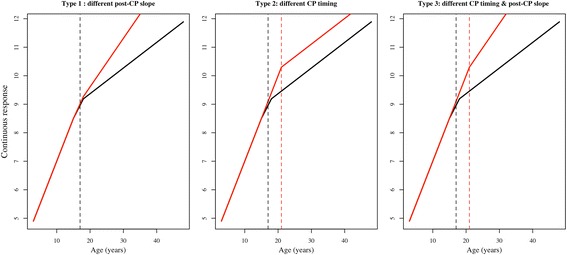
Three hypothetical models of between-group divergence in curvilinear response trajectories over time. Red and black solid lines indicate the average response curve of participants belonging to one or the other group; dashed lines show the position and age at change point(s) for the two groups of participants, or the age at which trajectories diverge between the two groups. Graph obtained using simulated data

The equation 2.2 above, allows for between-group difference in each of the 4 trajectory parameters of the piecewise model. (i.e. intercept, slope before and after the change point (CP), and the change point itself). If the focus is to determine and model the divergence in the trajectories between group, then model 2.2 can be modified by forcing the intercept and slope before the CP to be invariant across groups by setting *β*_0*grp*_ and *β*_1*grp*_ to zero at level 2 in equation 2.2. As illustrated in Fig. 1, we identify 3 possible ways in which continuous outcomes trajectories can diverge over time between groups: (1) type 1: the two groups have different slope after the CP, (2) Type 2: the two groups have different change points, and (3) Type 3: the two groups have different CP and post-CP slopes. To test for group-difference at different stages of the outcome development, our approach consists in fitting these 3 possible conditional Bayesian hierarchical models to the data and comparing model fit to determine which mechanisms provides the best representation of the underlying development of the outcome between groups of participants.

#### Bayesian estimation of the hierarchical piecewise model

We used a Bayesian approach to estimate and summarize the parameters of interest in the conditional multilevel piecewise model (formula 2.2) [86, 91]. In our illustrative example, all models were fit in RJAGS and R2JAGS in R.. In combined Bayesian notation, the trajectory model with a binary grouping status ‘GRP’ as the TIC covariate interacting with all 4 trajectory parameters can be written as follows:

$$ \begin{array}{l} Respons{e}_{i j}\sim Normal\left( m{u}_{i j},{\sigma}^2\right)\\ {} m{u}_{i j} = {v}_{0 i}+{\beta}_{0 grp} GR{P}_i + \left({v}_{1 i}+{\beta}_{1 grp} GR{P}_i\right) ag{e}_{i j} + \left({v}_{2 i}+{\beta}_{2 grp} GR{P}_i\right)\left( ag{e}_{i j}-\Big({v}_{CPi}+ C{P}_{grp} GR{P}_i\right)\Big){}^{+}\end{array} $$

To ensure that the effect of ‘group’ on each trajectory parameter can be either positive or negative and that the prior information does not dominate the likelihood, uninformative priors for the fixed group effects *β*_0*grp*_, *β*_1*grp*_, *β*_2*grp*_, *CP*_*grp*_ can be set as *N* ~ (0, 10^4^). In vector notation, the random effects *v*_*i*_ = (*v*_*oi*_, *v*_1*i*_ , *v*_2*i*_ , *v*_*cpi*_ )^*T*^ are assumed to follow a multivariate normal distribution with mean *β* and unstructured 4 x4 variance-covariance matrix *φ* as in 1.3, where *β* = (*β*_0_, *β*_1_, *β*_2_, *CP*)^*T*^, the vector of population means. Traditionally in Bayesian analysis for random effects, InvWishart(Σ,k) is used as a conjugate prior to the unknown variance-covariance matrix of multivariate normal distributions, where *Σ* is a positive definite inverse scale matrix of degree of freedom *k* [93]. Inverse-Gamma (λ_1_, λ_2_) is often used as the conjugate prior to the variance of univariate normal distribution (i.e. for mutually independent random effects, and model error variance *σ* ^2^). Alternative prior distributions may be used for level 2 variances of independent random effects or for the variance components of multivariate normal distributions [41, 92, 93].

#### S*ignificance of group-differences in trajectory parameters*

Testing for group-differences in trajectory parameters is equivalent to investigating the significance of the grouping covariates parameters at level 2 in the hierarchical change point model. In the Bayesian context, this is done by looking at the posterior probability density for the " *β*_*grp*_” parameters in 2.2. (i.e. *β*_0*grp*_, *β*_1*grp*_., *β*_2*grp*_.and *β*_*CP*_) of the estimated covariate parameters. For example, the effect of ‘GRP’ on each trajectory parameter is significant if the 95% Bayesian credible intervals (CI) of the estimated regressors (i.e. each “*β*_*grp*_”) exclude zero, in which case, the estimated “*β*_*grp*_” can be interpreted as the shifts in each trajectory parameter in one group compared to the other group [77, 92, 94].

#### Model convergence, fit and adequacy

The choice of the best model among the suite of candidate (conditional) Bayesian hierarchical models can be based on two criteria: (1) the deviance information criterion DIC [95, 96], an index of quality of fit that is commonly used for Bayesian model comparison [97], and (2), the Bayesian posterior predictive p-value (PP p-value), obtained through posterior predictive checking of the likelihood of each potential model (the sum of residuals was used as a as a discrepancy measure) [41].

### Illustrative data

We illustrate the application of the proposed Bayesian piecewise modeling approach by using it to investigate the divergence in child-to adult trajectories of BMI between participants who do and do not develop adult T2DM in a well-studied ongoing population-based prospective cohort, the Cardiovascular Risk in YFS [15]. Details on study design and on the collection of cardiovascular risk factors between 1980 and 2011 are published elsewhere [98] and summarized in Additional file 1.

In a previously published work on the YFS cohort, elevated BMI in children between 9 and 18 years was associated with an increased risk of developing T2DM in adulthood [87]. Additionally, a sex- and insulin-adjusted mixed model incorporating participants ages as a categorical variable, suggested that differences in average BMI values between those who do and those who do not develop adult T2DM tended to emerge during adolescence, becoming marginally significant from the age of 15 years onwards. In this approach, the between-group difference at each age groups was assessed by pairwise comparisons of the predicted marginal means (i.e. LS-means), and did not incorporate BMI trajectory information at the individual or population level. In contrast, the proposed hierarchical piecewise regression approach considers and makes full use of individual trajectory information to test for group-differences at specific stages of BMI development from childhood to adulthood. Unlike categorical approaches, the proposed growth model provides a clearer representation of the underlying pathological BMI development among those who develop T2DM in adulthood.

In our illustration, we include 2540 YFS participants (1401 females and 1139 males) followed-up a maximum of six times between 1980 and 2011 (Additional file 2: Table S1). Information on adult T2DM status was collected on participants at their latest individual adult follow-up (i.e. dichotomous outcome coded 0 for participants without T2DM, and 1 for those with T2DM in 2001, 2007, or 2011). Included participants had at least one BMI measure available in childhood (i.e. in 1980, 1983 or 1986 between age 3 and 18 years). Participants had on average 4.98 repeated measures of BMI over the study period, with 90.7% of participants having 4 or more BMI measures (Additional file 2: Figure S1). 88 included participants (3.5%, 44 females and 44 males) had T2DM in adulthood. We excluded BMI observations made among those aged 3 years in 1980 so that the ages of participants considered in the trajectory analysis ranged from 6 to 49 years. 3 year olds were not included in the analysis since only 3 participants in this birth cohort developed T2DM in adulthood. Furthermore, the lack of BMI measures between 3 and 6 years, prevented modeling the downwards slope from infancy peak, nor the age at adiposity rebound, which usually occurs before age 6 years in normal weight children [99, 100]. Using BMI data collected on participants aged 6 years and over, we expect that most participants had reached this important childhood milestone, and that a linear trend was thus an appropriate functional form to approximate childhood BMI growth from that age (Additional file 3: Figure S1).

Since sex differences in childhood growth and pubertal timing have been demonstrated [101, 102], subsequent BMI trajectory modeling between age 6 and 49 years was conducted among males and females separately [103]. BMI, especially in adulthood, is slightly right skewed, but using log10 transformed BMI in the modeling approach presented below did not alter our conclusions. For ease of interpretation, we thus present results using untransformed BMI only.

Visual inspection of the sex-specific smoothed BMI trajectories confirms the presence of a divergence between the two groups in adolescence (Additional file 3: Figure S2). Compared to participants who remain healthy, those who develop T2DM seem to have greater average BMI levels by the time they are young adults, although it is unclear whether this divergence results from a group-difference in the timing at which the transition to a slower BMI growth rate happens (Type II divergence) from a group-difference in rate itself after puberty (Type I divergence), or from both (Type III divergence).

Although the distal outcome of ‘adult T2DM’ is the grouping factor of interest in our illustrative trajectory divergence analyses, we also demonstrate how the same modeling approach can be used to investigate potential inter-cohort variation in childhood to adulthood BMI trajectories by considering models with ‘year of birth’ as a categorical level 2 predictor of each of the 4 trajectory parameters. Individual age- and sex-specific BMI Z-scores at the first clinic (in 1980) were also included as level 2 predictors of each BMI trajectory parameters to investigate if systematic deviation from participants of comparable age and sex at baseline had any influence on the development of BMI trajectories later in life. All continuous covariates used in the analyses were standardized in order to stabilize the variance, improve normality of errors and linearity of the mean.

Specific values for the hyperparameters used in our illustrative analyses are given in Additional file 4. While in principle *φ* can be unstructured, in our application, convergence for some parameters could not be reached when considering an unrestricted covariance structure between all four random effects in the unconditional change point model (equations 1.1 and 1.2), probably due to over parameterisation. Because initial analyses suggested a correlation between the slopes before the change point (b_*1i*_ *)* and the difference in slopes after the change point (b_*2i*_), we constrained the model by including a non-zero correlation between these two random effects but setting independence for all other random effects, leading to a block diagonal structure *of φ* (Additional file 4). Based on DIC, this covariance structure was preferred over mutually independent random effects for both males and females (Additional file 4), and used when expending the trajectory models with level 2 predictors. In our application, we investigated prior sensitivity by fitting the unconditional BMI trajectory model using three sets of priors for the hyperparameters (Additional file 4). Because we found that the choice of hyperparameters had a minor influence on the marginal posterior distributions, for subsequent (conditional) analyses, we chose to report posterior estimates of parameters estimated from the set of priors that yielded the lowest DIC in the sensitivity analyses (Additional file 4). In this set the priors for the means of the change points were based on the sex-specific estimates that maximized the profile log likelihood for the fixed (population-average) breakpoints in the unconditional model (estimated at 16 years for females and 22 for males, see estimation method in Additional file 5). Using these priors for the change point means also kept computation running times reasonable.

For the other participants varying variable included in the analysis, sex- and age-specific BMI z-scores at the first visit and birth cohort, priors were set to N ~ (0,0.001) for all corresponding parameters (i.e. all *β*_*cohort*_ priors and *β*_*initialBMI* − *z* − *score*_). To remain consistent with previous analyses of this data set [87], time-varying measures of fasting insulin were log-transformed and standardized before being included as a level-1 predictor in the Bayesian hierarchical models to improve right skewedness and to linearize its relationship with BMI About 17% of the insulin measures were not available in the data. The missing data mechanism for ‘insulin’ was considered non-informative, as we have no reason to believe that the probability of an individual insulin measure being missing depends on the true value of this missing insulin observation (although it may be related to other observed variables for that individual). We thus consider that insulin is missing at random (MAR), and we specify a prior for this covariate [104]. Since *log (insulin)* is approximately normally distributed, we specify a *N* ~ (*μ*_log(*insulin*)_, *τ*_log(*insulin*)_ ) likelihood for *log(insulin)*_*i*_ and place a vague prior on its variance (i.e. *τ*_log(*insulin*) ~ *Gamma*(0.001,0,001)_ ). Under this parametrization, the posterior predictive distribution for *μ*_log(*insulin*)_ and *τ*_log(*insulin*)_ will be informed by the observed part of the data only. Although individual insulin measurements change at each data collection point, by adding *log(insulin)* as a level 1 covariate in the multilevel model, the estimated relationship between insulin and BMI development remains constant across time [45]. This is a reasonable assumption in our application, since data exploration did not suggest any systematic patterns of change in insulin levels at the intra-individual level as people age. That is, the age smoother estimate obtained by fitting a generalized additive mixed model had an estimated degree of freedom (*edf*) close to 1 and was not significant (*p-value* >0.3), which did not suggest a non-linear relationship between *log(insulin)* and age [105].

Approximate posterior distributions of the parameters of the models considered throughout the analyses are obtained via MCMC simulations. Each model ran with 4 independent parallel chains of the Gibbs sampler (see Appendix 3 for an example of code). For each model, the first 50000 iterations were discarded in a burn-in run, and the draws from the posterior were thinned by a factor of 10 to reduce serial correlation of the chains. The following 20000 iterations were used to obtain posterior distributions of model parameters by mixing the 4 sequences. Model convergence was assessed through MCMC iterations traceplots and Gelman-Rubin diagnostic [92], and residual errors were plotted to confirm they approximately followed a normal distribution.

## Results

### Divergence of BMI profiles in T2DM and non-T2DM YFS participants

Following the modeling approach presented in the Methods and the priors and their corresponding hyperparameters (Additional file 4: Table S1) we fitted the following set of conditional Bayesian hierarchical piecewise models for each sex: unconditional (Model A), adult T2DM status adjusted intercept (Model B), adult T2DM status adjusted childhood slope (Model C), adult T2DM status adjusted adult slope (Model D), adult T2DM status adjusted change point (CP) (Model E), adult T2DM status adjusted CP and adult slope (Model F), adult T2DM status adjusted change point, childhood and adult slopes (Model G), adult T2DM status adjusted intercept, and change point (Model G), and a model with all four parameters adjusted for adult T2DM status (Model H). As mentioned above, previous research on this data set suggested BMI levels were not significantly different between the two groups in childhood [87]. Models C (i.e. group difference in childhood slopes) and B (i.e. BMI response consistently higher in one group across the life course) were thus fitted to demonstrate our modeling approach. An annotated extract showing the RJAGS code syntax used to fit Model E is available in Additional file 6.

For both sex, the lowest DIC was obtained when fitting model E, which was also the best fitting model with PP p-values close to 0.5 (Table 1). This supported the type II divergence mechanism where a difference in BMI levels emerged between the two groups due to a group difference in the change point timing. BMI growth rate in adulthood for both sexes was decreased by two-thirds compared to childhood (i.e. 0.67- vs. 0.18 -, and 0.61- vs. 0.15 kg/m^2^ per year in childhood and adulthood for females and males respectively), and participants who developed T2DM had similar BMI yearly rates in adulthood compared to those who remained healthy (β_2T2DM_ effect not significant in model F for both sex Table 2). However, females who developed T2DM reached their developmental transition in BMI rate on average 12.37 years later (Table 2).

**Table 1:** Analyses of the divergence in BMI trajectories between T2DM adults and non-T2DM adults: assessment of Bayesian model complexity (effective number of parameters pD), and fit (deviance information criteria DIC) for each candidate model

	Model	Females	PP p-val	Males	PP *p*-val
Unconditional	A	26910 (2544)	0.47	19837 (2223)	0.55
T2DMgroup (int β_0_)	B	26670 (2366)	0.45	19741 (2270)	0.43
T2DMgroup (childhood slope β_1_)	C	26780 (2510)	0.6	19865 (2247)	0.58
T2DMgroup (Adulthood slope β_2_)	D	26701 (2401)	0.58	19828 (2242)	0.62
**T2DMgroup (change point CP)**	**E**	**26076 (2777)**	**0.52**	**19762** (2213)	**0.51**
T2DMgroup (CP + β_2_)	F	26504(2430)	0.6	19860 (2271)	0.54
T2DMgroup (CP + β_0_)	G	26436 (2751)	0.55	19896 (2242)	0.45
T2DMgroup (all 4 parameters_)_	H	26532 (2978)	0.52	19920 (2435)	0.51

Reported for each model are DIC (pD), and posterior predictive *p*-values (PP *p*-val). Best fitting models are indicated in bold characters

**Table 2:** Posterior mean parameter estimates for Bayesian hierarchical Piecewise BMI trajectory for best fitting trajectory divergence models in males and females (Models E)

		Females Model E	Males Model E
β_0_	I	26.5 (0.20)	27.46 (0.16)
β_1_	S1	0.67 (0.012)	0.61 (0.01)
β_2_	S2	−0.49 (0.015)	−0.46 (0.06)
CP	CP	16.02 (0.29)	21.62 (0.42)
CP _T2DM_	CP	12.37 (1.21)	6.47 (1.23)
σ_β0_		2.07 (0.05)	2.36 (0.07)
σ_β1_		0.02 (0.005)	0.06 (0.004)
σ_β2_		0.07 (0.006)	0.05 (0.004)
*σ*_*β*1*β*2_		0.11 (0.05)	0.14 (0.03)
*σ*_*CP*_		3.1 (0.26)	4.3 (0.2)
*σ*		1.33 (0.02)	1.21 (0.01)
*β*_log(insulin)_		1.01 (0.04)	0.98 (0.03)

Posterior standard deviations (uncertainty in the parameters) are reported in brackets. Reported β_0_ coefficients are in kg/m^2^, β_1_ and β_2_ are in kg/m^2^ per year, CP and CP _T2DM_ are in years. All σ coefficients are standard deviations for the corresponding growth parameters and the residual error. *β*_log(insulin)_ coefficients are in kg/m^2^ for a 1 sd increase in *log(insulin*) level

Similarly for males, estimated BMI growth rates were not markedly different between the two T2DM groups in childhood or in adulthood, and comparable to those estimated in females (Table 2). But again, compared with healthy adults, those who developed T2DM reached their slower BMI growth rate on average 6.47 years later.

The effect of the time-varying covariate of insulin at level 1 was significant for both males and females, with a 1-sd increase in *log(Insulin)* resulting in a BMI observation increased by 2.6 and 2.8 kg/m^2^ respectively (i.e. exp(*β*_log(insulin)_), Table 2). To assess potential differences in the magnitude of the insulin effect as a function of between-person characteristics, we expanded model E by including an interaction between ‘adult T2DM status’ and *log(insulin)*. For each sex, the estimated parameters were not significant (95% CI included 0), suggesting that the effect of insulin on BMI was homogenous between the two groups and across genders.

The estimates of the variance-covariance parameters of model E showed that the correlation between an individual’s BMI growth rate in childhood and adulthood is equal to 0.61 for females and 0.47 for males, suggesting that children who have greater yearly BMI increase rates also have greater adult rates of increase (correlation estimated as: $$ \raisebox{1ex}{${\sigma}_{\beta 1\beta 2}$}\!\left/ \!\raisebox{-1ex}{$\sqrt{\sigma_{\beta 1}^2*{\sigma}_{\beta 2}^2}$}\right. $$, Table 2). The between-participant variation around the change point *σ*_*CP*_ was comparable between males and females (Table 2).

**Fig. 2:**
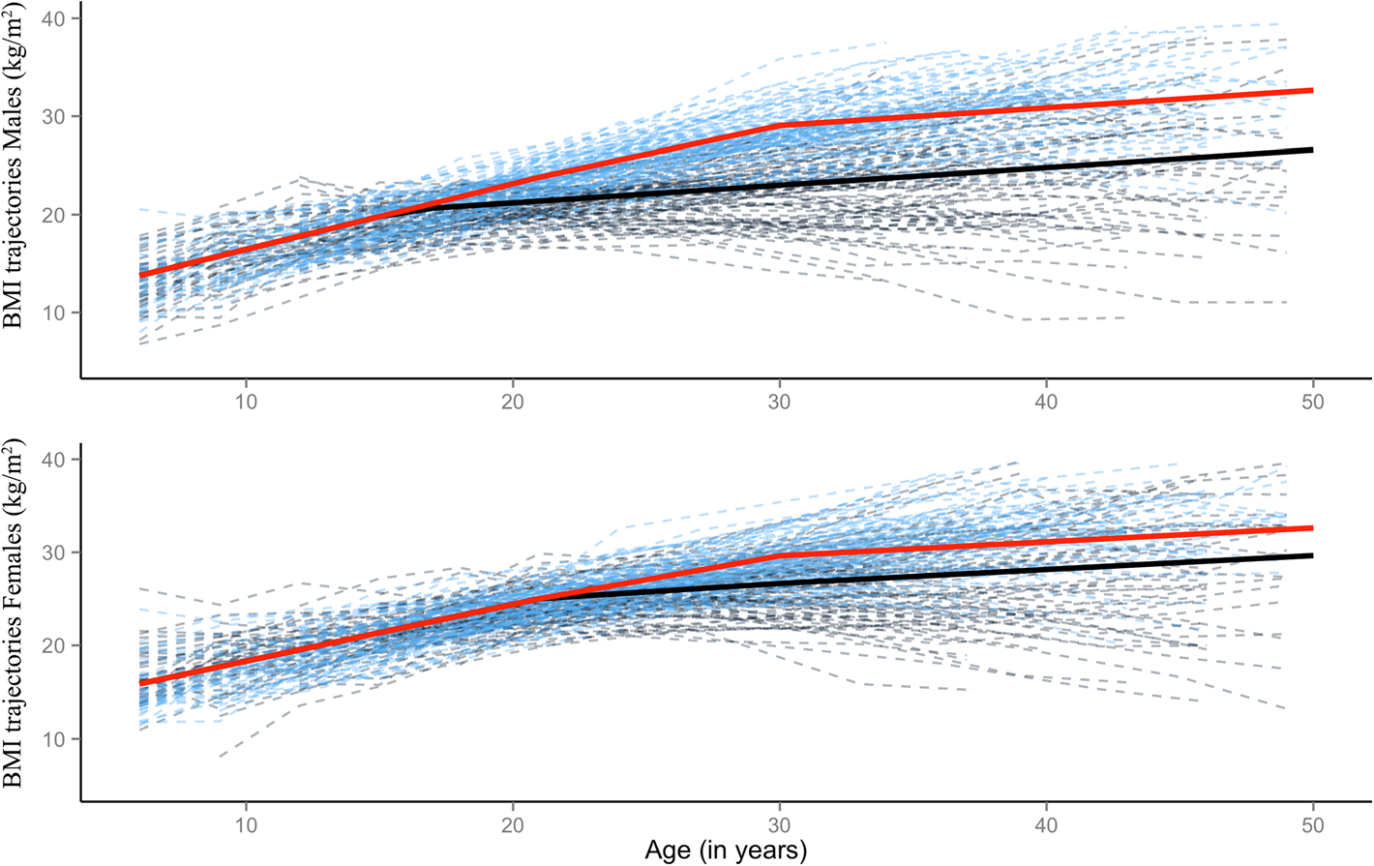
Sex-specific population average prototypical BMI trajectories for healthy and T2DM adults in the YFS cohort (solid blue and solid red lines, respectively) and prediction of 200 individual trajectories for each sex (100 per T2DM status group). The dashed trajectories were obtained by MCMC simulation using sex-specific posterior estimates of mean and variance of growth parameters for the best fitting models (Model E). In these predictions, time varying measures of *log(insulin)* were set to the average log(Insulin) observed in the cohort

**Fig. 3:**
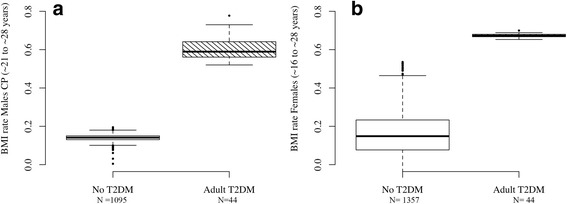
Box and whiskers plot of fitted individuals random slopes between 16.02 and 28.4 years for females (**a**) and between 21.62 and 28.09 years for males (**b**). Individual random slopes are estimated from the Bayesian hierarchical random change point model E. Solid lines in the boxplot indicate the group-specific median for the slopes (equivalent to the 50th percentiles of the posterior distribution)

Figure 2 shows the estimated population-average prototypical trajectories for each sex and T2DM group obtained from the estimated parameters for Model E, along with 100 trajectories predicted for each sex and T2DM group from Model E by Monte Carlo simulation. This illustrates a range of credible individual profiles generated under this model (see Appendix for code). For each sex and adult T2DM status group, Fig. 3. shows a box and whiskers plot of the estimated individual BMI slopes obtained from Model E after the average change point in the healthy group and before the T2DM groups reach their average CP (i.e. slopes between 16.02 and 28.4 years in females, and slopes between 21.62 and 28.09 years in males). Figure 3 illustrates that individual rates of change after puberty provides better discrimination of participants who went on to develop T2DM from those who did not, compared to punctual individual BMI levels at age 15 or 18 for females, and ages 21 and 24 for males (Additional file 2: Figure S2). While the distribution of BMI levels at age groups surrounding the age at divergence overlaps considerably (Additional file 2: Figure S2), individual slopes allow to differentiate participants who have switched to a rate consistent with a normal slowing down of BMI development after puberty, from those who are still on the trajectory of increasing BMI development consistent with the rate from childhood.

#### Effect of age-and sex-specific childhood Z-score on BMI trajectories

When including individual age-and sex-specific BMI z-scores at the first clinic as continuous level 2 predictors of each of the four growth parameters in sex-specific Models E, the only significant effect observed was for the childhood BMI slope, with a 1-sd increase in BMI z-score associated with a 0.056 (sd = 0.012) and a 0.038 (sd =0.009) increase in childhood (in kg/m^2^ per year) for male, and females respectively. This suggests that in the YFS sample, higher age- and sex-adjusted BMI at first visit in childhood were associated with faster BMI increase in childhood, but not with the age at transition in BMI development nor the change rate in adulthood.

#### Between cohort heterogeneity in BMI trajectories

To test whether ‘year of birth’ was associated with between-participant heterogeneity in the development of BMI from age 6 to 49 years, five binary dummy variables identifying BMI observations of people born in different years (i.e. 62, 65, 71, 74 and 77) were introduced as level 2 predictors of BMI growth parameters in sex-specific models (with year 1971 as the reference level) (Table 3). Increasing the complexity of the model did not improve model fit for males, and the lowest DIC was obtained for the unconditional model (Model A, Table 3) suggesting that their life-course BMI trajectory is more stable across cohorts. For females, model E marks a significant improvement in model fit, suggesting that the most significant predictor of between-cohort variations resides in the timing of the CP, although the best model was obtained when adjusting for a cohort effect on both the adult BMI growth rate and change point. For each sex, the posterior mean parameter estimates of the best fitting model are presented in Additional file 7. The results show that most of the between cohort variation for females is due to slight trajectory differences in two specific birth cohorts: those born in 1968, who reached the transition to adult BMI growth rate on average 2.89 years later than the cohort (year of birth 1971), and those born in 1974, who had adult BMI yearly rates increased by 0.06 (e.g. adult slopes of 0.24 compared to 0.18 kg/m^2^ per year on average for the other 5 cohorts) (Additional file 7).

**Table 3:** Analyses of inter-cohort differences in BMI trajectories: assessment of Bayesian model complexity (effective number of parameters pD), and fit (deviance information criteria DIC) for each candidate model

	Model	Females	PP p-val	Males	PP p-val
Unconditional	A	26910 (2544)	0.72	**197837(2223)**	**0.52**
Birth cohort (int β_0_)	B	26811 (2455)	0.70	19872 (2232)	0.70
Birth cohort (childhood slope β_1_)	C	26759 (2489)	0.34	19849 (2175)	0.63
Birth cohort (Adulthood slope β_2_)	D	26645 (2358)	0.67	19857 (2263)	0.68
Birth cohort (change point CP)	E	26395 (2599)	0.60	19862 (2211)	0.63
**Birth cohort (CP and β**_**2**_ **)**	**F**	**26390 (2671)**	**0.49**	19877 (2255)	0.43
Birth cohort (CP, β_2_ and β_1_)	G	26783 (2775)	0.48	19945 (2342)	0.53

Reported are: DIC (pD), and posterior predictive *p*-values (PP *p*-val). Best fitting models for each sex indicated in bold characters. (Convergence was not reached for the most complex model where all 4-trajectory parameters (i.e.β_0,_ β_1,_ β_2,_ and CP) were adjusted for birth cohort effects)

### Simulations

**Fig. 4:**
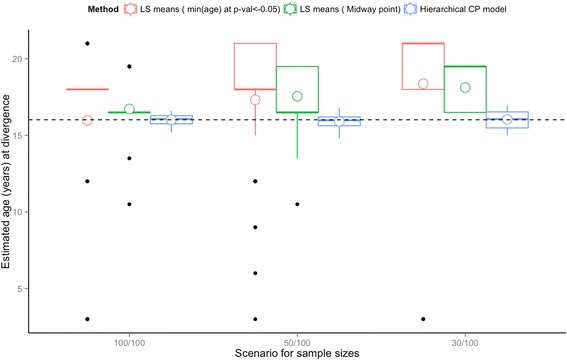
Boxplots and mean “Age at divergence” (x) estimated across 100 simulations using the three methods. Bottom and top of the boxes are the lower (*Q1*) and upper quartiles (*Q3*), respectively; the bands near the middle of the boxes are the medians, the lengths of the boxes represent the interquartile range (*IQR = Q3-Q1*); the upper whiskers are defined as min(max(x), Q3 + 1.5 * IQR) and the lower whiskers as max(min(x), Q1 – 1.5 * IQR). Means of age at divergence across the 100 simulations for each scenario are indicated with empty circles. The horizontal dashed line indicates the true age at divergence set in the simulations (16.02 years old)

A short series of simulations was conducted to compare difference in estimates of the age at which the groups diverge when using the proposed Bayesian piecewise growth modeling approach compared to a more traditional approach based on pairwise comparison of LS-means estimated from a categorical mixed model. We simulated repeated measure data from a Type II divergence model (i.e. group-difference in the change point timing only), using the posterior estimates of mean growth parameters for the model fitted for females (average parameters are set to: *β*_*O*_ = 26.5, *β*_1_ = 0.67, CP = 16.02, = *β*_*GroupCP*_ =12.37, *β*_2_ = −0.49, matching Model E posterior estimates for females in Table 2), and both a participant-level random effect (*σ*_*error*_^2^ = 2.77) and an observation-level residual error (*σ*_*error*_^2^ = 2.47). Under this model, “CP*”,* the change point for the first group to depart from the population-average childhood slope represents the age at which the two groups of participants diverge in their outcome trajectories (i.e. the second group maintains this rate of change for 12.30 years longer). To resemble the YFS BMI data, we randomly sampled baseline ages from the YFS cohort subtracted by 25 years as ages at the first visit for each participant, with 6 non-missing repeated measures 3, 6, 9, 21, 27 and 31 years later for each participant. We considered 3 scenarios of sample sizes for the number of participants in each group (group 1/group 2): (1) 100/100, (2) 50/100, and (3) 30/100. For each of the three scenarios, we simulated 100 datasets and fitted both a mixed model with age as a categorical variable and the Type II divergence Bayesian Hierarchical piecewise model using the set of priors defined in Additional file 4. For each piecewise model, we recorded the posterior estimate for the “CP” parameter, and for each fitted categorical mixed model, we applied pairwise comparison of the least-square means (LS-means) with Tukey adjustment for multiplicity to retrieve: (1) the earliest age at which the group-difference in means was found significant (*p* < 0.05), and (2) the midway point between two consecutive ages that had a minimum number of non-significant differences in means before and significant differences in means after the “midway point” method (2) is a potential alternative definition of age at which the group-difference appears in the LS means. Compared to the “earliest age with p-val <0.05” method (1), the “midway” point definition minimises the impact of simulations where some tests show significance at a young age, even though tests for the surrounding ages are not. For each scenario, estimated ages at divergence using the 3 methods were averaged across the 100 simulations. Figure 4 presents the simulation results in term of the quartiles distribution and means of these estimates of age at divergence across the 100 simulations. When sample size decreases for one group of participants, the pairwise LS mean comparison method will tend to overestimate the age at divergence, with significant variability in the estimates arising due to random variation, especially when age at significance is determined using the first age at which a p-value <0.05 occurs (Fig. 4.). In contrast, the hierarchical Bayesian piecewise model was less sensitive to sample size, and the true age at divergence was consistently within the estimated interquartile range of the produced estimates, indicating that the Bayesian trajectory divergence model outperforms the LS mean method in both accuracy and precision, regardless of the way “age at divergence” is defined from the model output.

## Discussion

Using the repeated BMI data from the YFS study, we demonstrated how Bayesian hierarchical piecewise regression (BHPR) modeling may be used to investigate between-group trajectory divergence in non-linear longitudinal outcomes.

The non-linearity in BMI development across the life-course is well documented in the literature, with changes in BMI corresponding to a number of identified developmental phases [101, 106, 107]. In particular, BMI rate decelerates after puberty once people reach their adult height, translating to a leveling-off of the BMI trajectory in adulthood [108, 109]. Although many recent applications have relied on such approaches [99, 102, 110–113], traditional polynomial parameterizations of growth curve models are not well suited to analyse BMI development [114, 115], especially if the focus is to identify transitional changes or determine divergence between groups.

In contrast, piecewise regression is particularly suited to model BMI across different life-stages as its parameters map onto what is known about the natural development of BMI over time [116]. Since ‘change points’ (i.e. milestones in the case of BMI) are model parameters in the piecewise model, there is no need to use elaborate techniques to retrieve these points of interest [59, 112, 117]. Piecewise models are also often preferable to more general continuous non-linear models if the number of repeated measurements per participant is small (i.e. 3 to 6 data points each as in [16, 109]) as is often the case in long-running observational prospective studies [99, 112]. Moreover piecewise multi-level regression models may be used to characterize the divergence mechanisms in non-linear responses between groups by modeling change points as random parameters and introducing grouping factors as predictors of the between-person heterogeneity in responses over time.

Although our main goal was to characterize how and when the developmental patterns of BMI diverged between those who did and did not develop T2DM in the YFS, we also demonstrated the utility of the method to investigate cohort effects in the outcome response. Previous analyses of the YFS BMI and T2DM data considered categorical mixed models and tested for differences in the estimated BMI levels between the two T2DM groups at different ages by pairwise comparisons of the BMI predicted marginal means (i.e. Least-Square means) averaged over sex while adjusting for multiple testing (i.e. Tukey adjustment). This approach suggested that from age 15 years, the T2DM group had significantly higher BMI levels than those without T2DM. However, these analyses ignored the potential confounding effect of birth cohorts, and each existing “age” was treated as a non time-ordered categorical variable so that no inference could be made on individual or group-specific age-related BMI trajectories. Some age groups comprised those from up to five separate birth cohorts, while others only comprised those from a unique birth cohort (i.e. those aged 3 and 27 years). Having substantially fewer participants in one or both T2DM status groups at some age points results in a decreased power to detect a significant difference between groups (i.e. the observed difference at age 27 years was not significant in either sex-averaged or sex-adjusted LS-means, Additional file 8: Tables S1, S2 and Figure S1, S2). Because BMI development is known to progress differently in males and females, and the oldest and youngest cohorts in the YFS sample are almost a generation apart (~15 years), not taking these confounders into account may result in biased inferences. In fact, when estimating the LS-means separately for each sex, the age at which the difference between T2DM groups becomes significant is not as clear since in males the difference is not significant at age 21 and 24 years, suggesting the true divergence in BMI between T2DM groups for males occurs more around those ages (Additional file 8: Table S2 and Figure S2).

In contrast, the method we illustrate here is not sensitive to sample size and uses developmental theory to inform a model that allows between-group differences in within-person BMI trajectories at four possible levels for males and females to be examined (i.e. the overall BMI level, the childhood BMI growth rate, the adult BMI growth rate, and the age at which the transition between the two phases of change occurs).

Applied to the example data set, our method allowed us to characterise group differences in the non-linear development of BMI and to identify a critical age window at which weight intervention programs might be best applied to help reduce or delay the incidence of T2DM in adulthood. Our findings support the theory that girls who keep on gaining weight at the same rate they did in childhood past the age of 16 years are more likely to develop T2DM in adulthood. Similarly, for males, the natural deceleration in BMI velocity occurs, on average, at 21 years of age. Those who stay on their childhood BMI trajectory past that age may be at increased risk of developing T2DM.

Longitudinal studies often aim to make inferences on differences among average population health marker trajectories. Typically, this involves comparing change rates (or slope differences) in healthy participants vs. those with pathological development, specific treatment conditions, or groups following certain lifestyle patterns [118–120]. Using our Bayesian hierarchical piecewise regression approach, serial measures of patient’s weight and height, often routinely collected in paediatric, general practice, and healthy or well child clinics, could be used to determine if an individual is on a path to an healthy adult weight status, or if their BMI trajectory places them in a category more susceptible to develop adult metabolic conditions such as T2DM.

## Conclusions

Studying within-person and between-person differences in the development of continuous outcomes as a function of age in long-running multi-cohort observational studies is crucial to better understand the natural history of healthy vs. pathological risk factor profiles. Due to the typically unbalanced data designs, loss to follow-up and expected non-linear responses, it remains methodologically challenging to analyse such data. When the substantial focus is on when and how two or more groups of participants grouped according to a distal dichotomous health outcome have diverged in their response trajectories, traditional parameterisations of curvilinear growth model do not allow to identify an age at which the group that developed the condition moved onto a different path compared to the group that remained healthy. In contrast, the hierarchical piecewise multi-level modeling enables the separation of multiple aspects of change in complex developmental processes such as individual and group differences in the rates of change at different periods, and potential heterogeneity in the timing at which individuals from identified groups enter each developmental phase, providing a powerful tool to help inform intervention The methodology we illustrate here focuses on a response with only one developmental change point, but it could easily be extended to more complex non-linear responses with multiple transitions.

## Additional files

Additional file 1: Additional information on BMI, T2DM status and fasting insulin information collection in the YFS subset used in the illustrative analyses. (DOCX 17 kb)

Additional file 2: Subset of the YFS cohort used for the BMI trajectory analysis. Reported are the total number (No.) of participants seen at each clinic year and their ages (**Figure S1.**) Density plot of the number of BMI measures per YFS participants in the subset of the cohort used for the BMI trajectory analysis (**Figure S2.**) and average BMI values in kg/m^2^ at each age stratified by T2DM group (pink, no adult T2DM; blue, Adult T2DM), with error bars representing the mean BMI ± SD (standard deviation) (**Figure S3.**) (DOCX 180 kb)

Additional file 3: Spaghetti plot of the individual trajectories of those with T2DM in adulthood (*N* = 88) and those who did not develop T2DM in adulthood (*N* = 2452). Red solid line: loess smoother curve indicating the average longitudinal trend in each group (**Figure S1.**) and scatterplot of the life-course BMI data (in kg/m^2^) stratified by sex. Solid lines and gray bands: loess smoothed average trajectories and confidence intervals for each group (adult T2DM vs. non-T2DM group); dashed lines: age-specific averages of BMI levels (**Figure S2.**) (DOCX 1918 kb)

Additional file 4: Prior sensitivity analyses methods and results. (DOCX 25 kb)

Additional file 5: Log-likelihood profiling method and R-code for the choice of priors of the changepoint mean. (μ_cp). (DOCX 14 kb)_

Additional file 6: Annotated RJAGS sample code to fit a type 1 trajectory divergence model with a fully unstructured 4 by 4 covariance matrix for the random growth parameters. (DOCX 14 kb)

Additional file 7: Posterior mean parameter estimates for best fitting birth cohort-adjusted trajectory model for each sex (Model E for females and Model A for males). (DOCX 16 kb)

Additional file 8: Results of mixed models with age as a categorical predictor and log-insulin as a continuous predictor: LS means contrasts (No adult T2DM vs. adult T2DM) and significance at each age averaged over- (**Table S1.**) or adjusted for the levels of sex (**Table S2.**) and pairwise comparisons of Least-square means of BMI and 95% CIs at each age in each T2DM status group averaged over levels of sex (**Figure S1.**) at each age in each T2DM status group and sex group combination (M = males, F-females, 1 = No adult T2DM, 2 = adult T2DM) (**Figure S2.**) and adjusted for *log(insulin). (DOCX 549 kb)*

## Abbreviations

BHPR: Bayesian hierarchical piecewise regression
BMI: Body mass index
CI: Credible interval
CP: Change point
CVD: Cardiovascular disease
DIC: Deviance information criteria
MCMC: Markov Chain Monte Carlo
PP p-value: Posterior predictive p-value
T2DM: Type 2 Diabetes mellitus
TIC: Time-independent covariate
TVC: Time-varying covariate
YFS: Cardiovascular risk in Young Finn study
