# Supplementary material for: Bayesian hierarchical piecewise regression models: a tool to detect trajectory divergence between groups in long-term observational studies
Source: BMC Med Res Methodol. 2017 Jun 6;17:86. doi: 10.1186/s12874-017-0358-9 (PMC5461770; doi:10.1186/s12874-017-0358-9)
Supplement: Supplementary file 1 — Additional information on BMI, T2DM status and fasting insulin information collection in the YFS subset used in the illustrative analyses. (DOCX 17 kb) [file 12874_2017_358_MOESM1_ESM.docx]

**Additional file 1.**

**Additional information on BMI, T2DM status and fasting insulin information collection in the YFS subset used in the illustrative analyses.**

The Cardiovascular Risk in Young Finn Study is an ongoing population-based prospective cohort that started in Finland in 1980 to investigate cardiovascular risk factors and their determinants from childhood to adulthood, with participants ranging from age 3 to 49 [5]. Between 1980 and 2011, along with other cardiovascular markers of interest, BMI, calculated as BMI = weight (kg) / [height (m)]^2^, was collected on a varying number of participants from the original cohort (consisting of 3596 children aged 3 to 18 years in 1980), with up to 6 follow-up measurements per participants: 3 in childhood/young adulthood, and 3 in middle adulthood (Table 1 in S2 File). Fasting Insulin was also measured up to 6 times per participants using methods as previously described [10].

For the BMI calculations, standing height was measured to the nearest 0.5 cm using a wall-mounted Seca anthropometer, with the participant in bare feet. Weight was measured with participants in light clothes without shoes using bathroom digital Seca weighing scale that recorded to the nearest 0.1 kg.

T2DM status in adulthood was determined at any adult follow-ups (2001, 2007, and 2011) as any of: a fasting plasma glucose ≥7 mmol/L (in the absence of type 1 diabetes mellitus), a hemoglobin A1c ≥6.5% (48 mmol/mmol), self-reported in questionnaires of a diagnosis made by a physician, or currently taking an oral glucose-lowering medication. In the analysis, T2DM was used as a dichotomous outcome (i.e. dummy variable coded 0 for participants who remained healthy, and 1 for those with T2DM at their latest adult follow-up (either 2001, 2007 or 2011).

Participants or their parents provided informed consent, and the study was approved by local ethics committees in agreement with the Declaration of Helsinki.
