# Supplementary material for: Bayesian hierarchical piecewise regression models: a tool to detect trajectory divergence between groups in long-term observational studies
Source: BMC Med Res Methodol. 2017 Jun 6;17:86. doi: 10.1186/s12874-017-0358-9 (PMC5461770; doi:10.1186/s12874-017-0358-9)
Supplement: Supplementary file 2 — Subset of the YFS cohort used for the BMI trajectory analysis. Reported are the total number (No.) of participants seen at each clinic year and their ages (Figure S1.) Density plot of the number of BMI measures per YFS participants in the subset of the cohort used for the BMI trajectory analysis (Figure S2.) and average BMI values in kg/m2 at each age stratified by T2DM group (pink, no adult T2DM; blue, Adult T2DM), with error bars representing the mean BMI ± SD (standard deviation) (Figure S3.) (DOCX 180 kb) [file 12874_2017_358_MOESM2_ESM.docx]

**Additional file 2**

**Subset of the YFS cohort used for the BMI trajectory analysis**

**Table S1.** The total number (No.) of participants seen at each clinic year and their ages

| **Year** | **No.** | **YOB^a^** | 77 | 74 | 71 | 68 | 65 | 62 |  |  |  |  |  |  |  |
| --- | --- | --- | --- | --- | --- | --- | --- | --- | --- | --- | --- | --- | --- | --- | --- |
| 1980 | 2149 | **Ages** | 3* | 6 | 9 | 12 | 15 | 18 |  |  |  |  |  |  |  |
| 1983 | 2149 |  |  | 6 | 9 | 12 | 15 | 18 | 21 |  |  |  |  |  |  |
| 1986 | 1970 |  |  |  | 9 | 12 | 15 | 18 | 21 | 24 |  |  |  |  |  |
|  |  |  |  |  |  |  |  |  |  |  |  |  |  |  |  |
| 2001 | 2167 |  |  |  |  |  | 24 | 27 | 30 | 33 | 36 | 39 |  |  |  |
| 2007 | 2058 |  |  |  |  |  |  | 30 | 33 | 36 | 39 | 42 | 45 |  |  |
| 2011 | 1890 |  |  |  |  |  |  |  | 34 | 37 | 40 | 43 | 46 | 49 |  |

**^a^:**YOB (year of birth), corresponds to the 6 birth cohorts represented in the study sample.

*: Observations made on 3 years old were excluded from the analyses).

**Figure S1.** Density plot of the number of BMI measures per YFS participants in the subset of the cohort used for the BMI trajectory analysis.


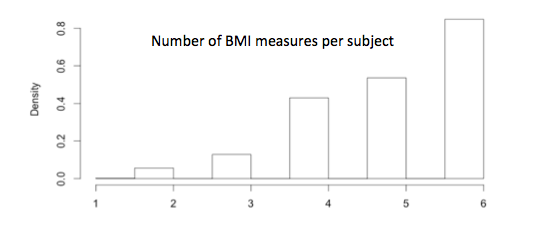


**Figure S2.** Average BMI values in kg/m^2^ at each age stratified by T2DM group (pink, no adult T2DM; blue, Adult T2DM), with error bars representing the mean BMI ± SD (standard deviation).

**
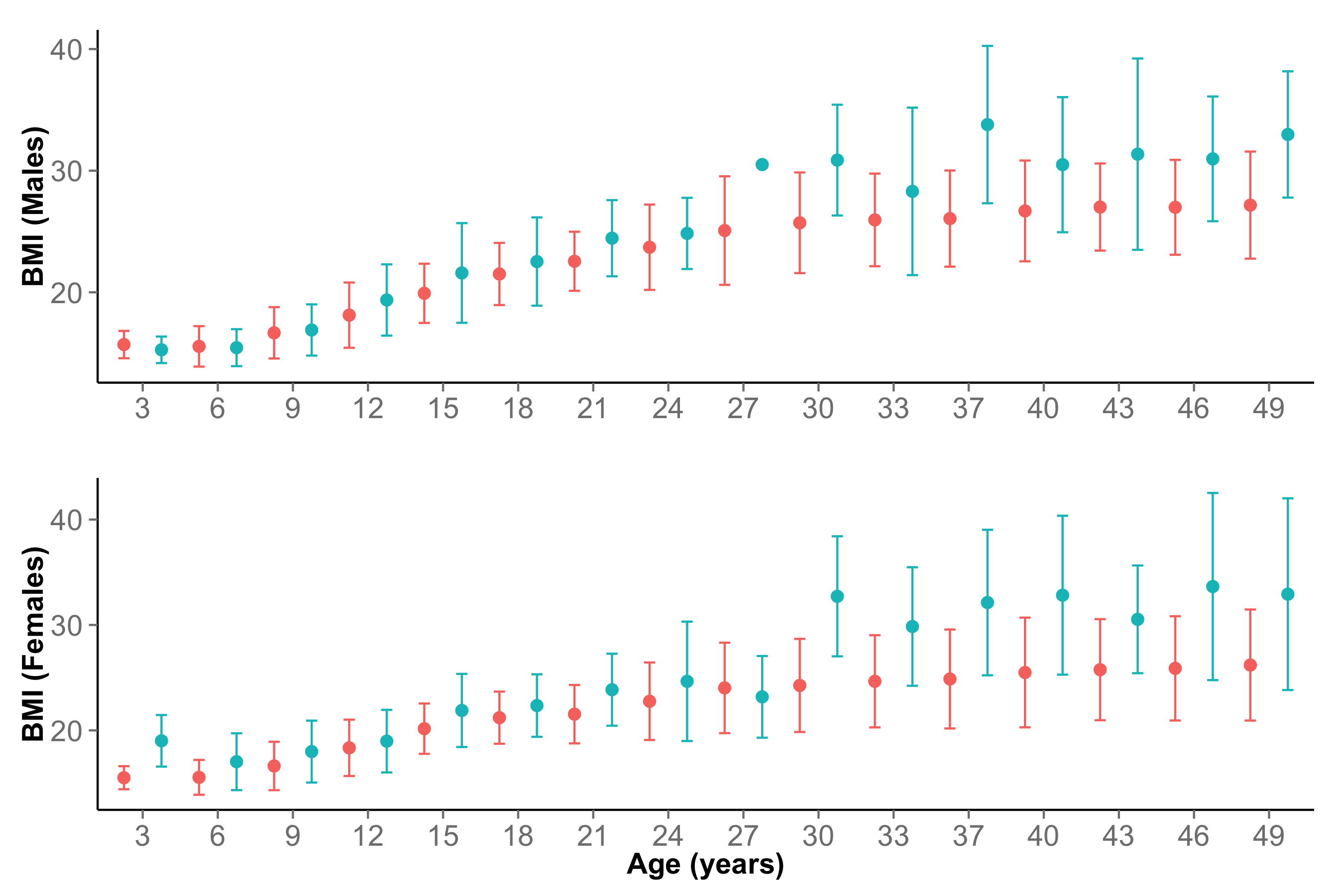
**
