# Supplementary material for: Bayesian hierarchical piecewise regression models: a tool to detect trajectory divergence between groups in long-term observational studies
Source: BMC Med Res Methodol. 2017 Jun 6;17:86. doi: 10.1186/s12874-017-0358-9 (PMC5461770; doi:10.1186/s12874-017-0358-9)
Supplement: Supplementary file 3 — Spaghetti plot of the individual trajectories of those with T2DM in adulthood (N = 88) and those who did not develop T2DM in adulthood (N = 2452). Red solid line: loess smoother curve indicating the average longitudinal trend in each group (Figure S1.) and scatterplot of the life-course BMI data (in kg/m2) stratified by sex. Solid lines and gray bands: loess smoothed average trajectories and confidence intervals for each group (adult T2DM vs. non-T2DM group); dashed lines: age-specific averages of BMI levels (Figure S2.) (DOCX 1918 kb) [file 12874_2017_358_MOESM3_ESM.docx]

**Additional file 3**

**Figure S1.** Spaghetti plot of the individual trajectories of those with T2DM in adulthood (N=88) and those who did not develop T2DM in adulthood (N=2452). Red solid line: loess smoother curve indicating the average longitudinal trend in each group.

**
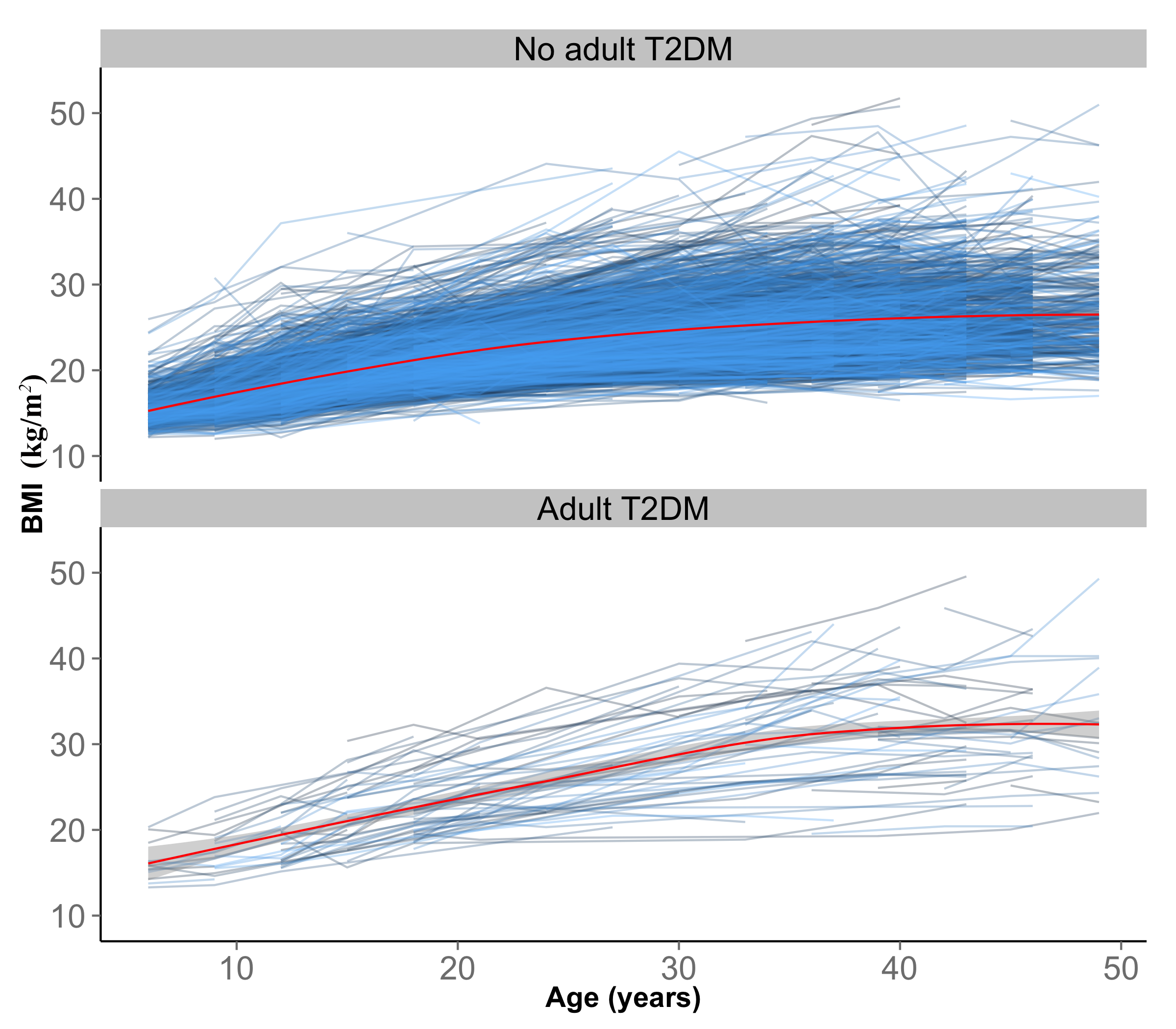
**

**Figure S2.** Scatterplot of the life-course BMI data (in kg/m^2^) stratified by sex. Solid lines and gray bands: loess smoothed average trajectories and confidence intervals for each group (adult T2DM vs. non-T2DM group); dashed lines: age-specific averages of BMI levels.

**
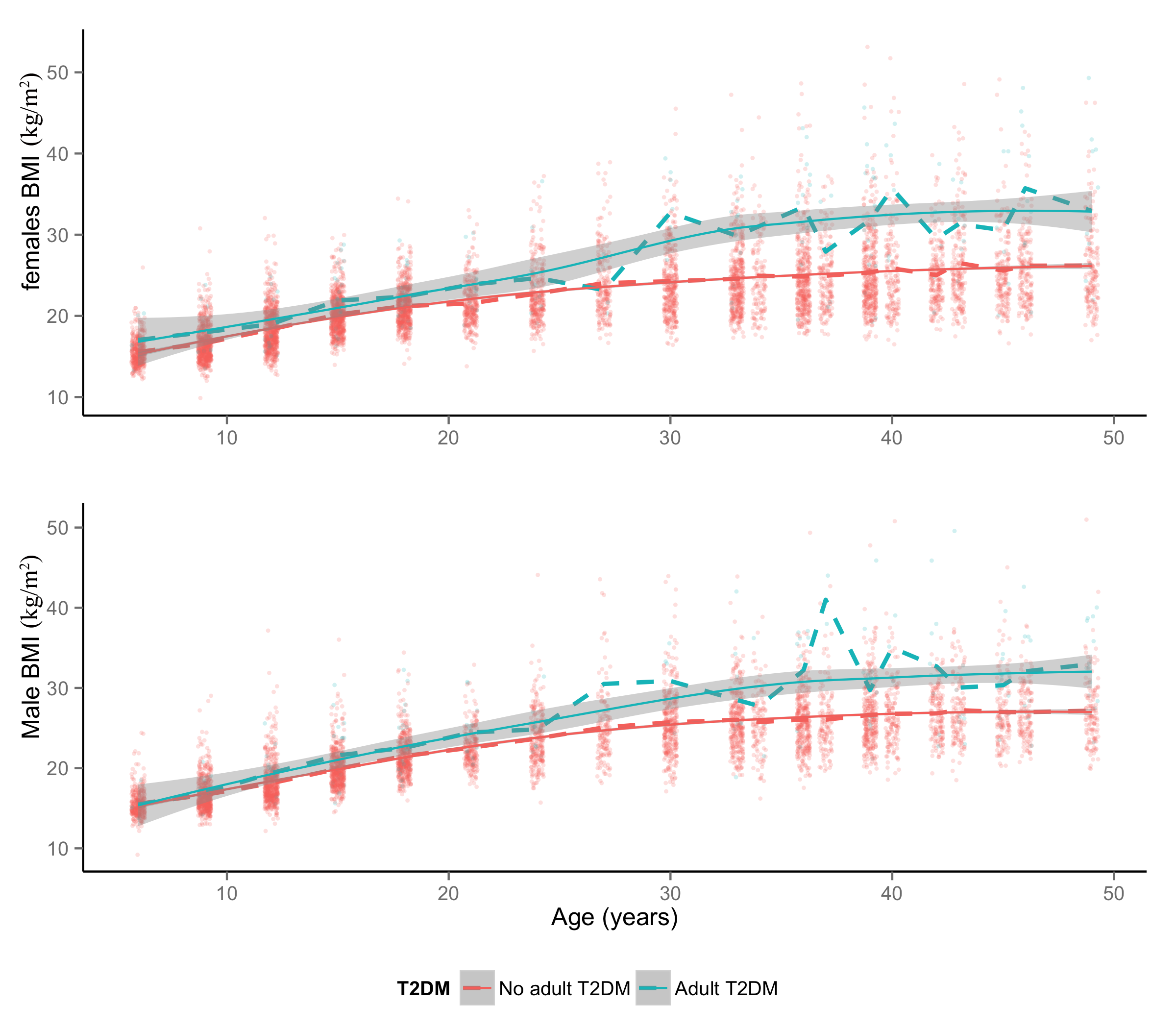
**
