# Supplementary material for: Bayesian hierarchical piecewise regression models: a tool to detect trajectory divergence between groups in long-term observational studies
Source: BMC Med Res Methodol. 2017 Jun 6;17:86. doi: 10.1186/s12874-017-0358-9 (PMC5461770; doi:10.1186/s12874-017-0358-9)
Supplement: Supplementary file 4 — Prior sensitivity analyses methods and results. (DOCX 25 kb) [file 12874_2017_358_MOESM4_ESM.docx]

**Additional file 4.**

**Prior sensitivity analyses methods and results.**

Box summarizing priors and hyper priors used in the conditional trajectory models analyses. (Note that priors listed for $\beta_{grp}$ refer to both $\beta_{T2DMgrp}$ and all $\beta_{cohort}$ in the analyses).

**Second stage:** Priors for random effects

$$\left( \begin{aligned} b_{1i} \\ b_{2i} \end{aligned} \right)|\left( \begin{aligned} \beta_{1} \\ \beta_{2} \end{aligned} \right)\sim MVN\left\{ \left( \begin{aligned} \beta_{1} \\ \beta_{2} \end{aligned} \right),\Sigma_{\beta1\beta2} \right\}$$

$$b_{0i}|\beta_{0},\tau_{b0} \sim N(\beta_{0},\tau_{b0})$$

$${CP}_{i}|\mu_{CP},\tau_{CP} \sim N(\mu_{CP},\tau_{CP})$$

**Third stage:** Hyperpriors for population parameters

$$\left( \begin{aligned} \beta_{1} \\ \beta_{1} \end{aligned} \right)\sim MVN\left\{ \left( \begin{aligned} 0 \\ 0 \end{aligned} \right),\left( \begin{matrix} 10 & 0 \\ 0 & 10 \end{matrix} \right) \right\}$$

$$\Sigma_{\beta1\beta2}\sim\mathrm{invWishart}\left\{ \left( \begin{matrix} 0.1 & 0 \\ 0 & 0.1 \end{matrix} \right),2 \right\}$$

$\beta_{0} \sim N(25,0.001)$ and $\tau_{b0} \sim\mathrm{Gamma} (0.01,0.01)$

$\mu_{CP} \sim N(16,0.001)$ (Females) or $\mu_{CP} \sim N\left( 22,0.001 \right)(Males)$ and $\tau_{CP} \sim G\mathrm{amma}(0.01,0.01)$

All $\beta_{grp}\sim N(0,0.001)$ (i.e. $\beta_{0grp},\beta_{1grp},\beta_{2grp}{,CP}_{grp}$for each growth parameter)

$\beta_{initialBMI-z-score}$ $\sim N(0,0.001)$

And $\varepsilon_{ij}|\tau_{i} \sim N(0,\tau_{i})$ &$\tau_{i} \sim\mathrm{Gamma} (0.01,0.01)$

**Prior sensitivity analysis**

Convergence could not be reached for some parameters in initial analyses of the sex-specific unconditional hierarchical BMI changepoint models with unrestricted covariance structure between all 4 random parameters (Males and females BMI profiles were analysed separately). As explained in section 3,we chose to restrict each model with a correlation between individual childhood slopes ($\beta_{1}$) and adult slopes ($\beta_{2}$), and null correlations between other random effects leading to a block diagonal structure of the variance covariance matrix:

$$\beta_{12}\sim MVN\left\{ \beta_{12}^{*},H_{12} \right\}$$

$$\beta_{0} \sim N(\beta_{0}^{*},H_{0})$$

$$\Sigma_{12}\sim\mathrm{invWishart}\left\{ \Sigma_{12}^{*},2 \right\}$$

$$\tau_{b0} \sim\mathrm{Gamma} (\lambda_{01},\lambda_{02})$$

$$\tau_{i} \sim\mathrm{Gamma} (\lambda_{1},\lambda_{2})$$

$$\tau_{CP} \sim\mathrm{Gamma} (\lambda_{CP1},\lambda_{CP2})$$

With:

$H_{0}$ the variances for the overall intercept (i.e BMI at 25 years), and $H_{12}$ the covariance matrix for $\beta_{12}=(\beta_{1.},\beta_{2}$). $\tau_{b0} \tau_{i} \tau_{CP}$ are the precisions (1/variance) for each corresponding parameter (i.e. variances are inverse gamma distributed). Sex specific unconditional models with this block diagonal structure yield lower AIC compared to models with mutually independent random effects, indicating this was an appropriate way to represent the variance structure of the random effects (results not shown here).

For the choice of priors for the changepoint means we considered 3 potential distributions:

- Normal (${CP}_{i} \sim N(\mu_{CP}^{*},H_{CP})$), with$\mu_{CP}^{*}$ and $H_{CP}$ the mean and variance for the changepoint, respectively. (we chose : $\mu_{CP}^{*}$ =0, $H_{CP}=1000$)
- Exponential (${CP}_{i} \sim Exp(\lambda)$), where the changepoint mean is 1/$\lambda$and the changepoint variance is1/$\lambda^{2}$. (we chose $\lambda$ $=0.05 (i.e.\frac{1}{17}$, lowest expected age at transition)
- Uniform (${CP}_{i} \sim uniform(a,b)$), where the mean is b+a/2 and the variance is (b-a)^2^/12. (we chose a= -16 (i.e 9-25 ),b=25 (i.e. 50-25), as ages range between 9 and 50 years and are centered around the grand mean age of 25 years)

The 2 sex-specific unconditional growth models were fitted with each of these 3 priors for the changepoint means ( and the priors above for the other model parameters). For both sex the lowest AICs were obtained for ${CP}_{i} \sim N(\mu_{CP}^{*},H_{CP})$), suggesting that the uniform and exponential distributions are not the best distributions for the changepoint means (results not shown). As a result, we only consider the prior $CP \sim N(\mu_{CP}^{*},H_{CP})$ for the changepoint means in all subsequent analyses.

Additionally, to investigate the sensitivity of the unconditional trajectory model’s results to the choice of the different hyperparameters, we consider three sets of hyperparameters:

| **Hyperparameter** | **Priors 1** | **Priors 2** | **Priors 3** |
| --- | --- | --- | --- |
| $\beta_{0}^{*}$ | 0 | 15 | 25 |
| $\beta_{12}^{*}$ | (0,0) | (0.5,0.2) | (0,0) |
| $\mu_{CP}^{*}$ | 0 for both sexes | 10 for both sexes | 16 for females  21 for males |
| $H_{0}$ | 10 | 100 | 100 |
| $H_{12}$ | $\left( \begin{matrix} 10 & 0 \\ 0 & 10 \end{matrix} \right)$ | $\left( \begin{matrix} 10 & 0 \\ 0 & 10 \end{matrix} \right)$ | $\left( \begin{matrix} 100 & 0 \\ 0 & 100 \end{matrix} \right)$ |
| $H_{CP}$ | 10 | 100 | 100 |
| $\Sigma_{12}^{*}$ | $\left( \begin{matrix} 10 & 0 \\ 0 & 10 \end{matrix} \right)$ | $\left( \begin{matrix} 1 & 0 \\ 0 & 1 \end{matrix} \right)$ | $\left( \begin{matrix} 0.1 & 0 \\ 0 & 0.1 \end{matrix} \right)$ |
| $\lambda_{01}$ | 0.01 | 0.1 | 0.001 |
| $\lambda_{02}$ | 0.01 | 0.1 | 0.001 |
| $\lambda_{CP1}$ | 0.1 | 0.01 | 0.001 |
| $\lambda_{CP2}$ | 0.1 | 0.01 | 0.001 |
| $\lambda_{1}$ | 0.1 | 0.01 | 0.001 |
| $\lambda_{2}$ | 0.1 | 0.01 | 0.001 |
| AIC Males | 20130 | 19995 | 19716 |
| AIC females | 28756 | 27405 | 26910 |

**Table S1** Three different sets of hyperparameters used for the prior sensitivity analysis

We found that the posterior distributions of the model parameters did not seem to have a lot of influence on the marginal posterior distributions of the unconditional models parameters (See Tables of estimated parameters below). We chose to use the set of priors 3 in all subsequent conditional analyses as they showed the lowest AIC, and also reduced computation time significantly.

| **Parameters**  **Females** | **Prior 1** | **Prior 2** | **Prior 3** |
| --- | --- | --- | --- |
| β_0_ | 27.25 (0.17) | 25.91 (0.47) | 26.45 (0.17) |
| β_1_ | 0.63 (0.01) | 0.58 (0.03) | 0.61 (0.01) |
| β_2_ | -0.49 (0.014) | -0.43 (0.01) | -0.45 (0.014) |
| CP | 16.71 (0.2) | 15.95 (0.3) | 16.01 (0.2) |
| $\sigma_{\beta0}$ | 2.44 (0.05) | 2.68 (0.06) | 2.12 (0.05) |
| $\sigma_{\beta1}$ | 0.026 (0.005) | 0.014 (0.004) | 0.02 (0.003) |
| $\sigma_{\beta2}$ | 0.15 (0.005) | 0.16 (0.007) | 0.19 (0.005) |
| $\sigma_{CP}$ | 3.34 (1.14) | 4.79 (1.21) | 3.12 (1.07) |
| $\sigma_{\beta1\beta2}$ | 0.09 (0.24) | 0.14 (0.25) | 0.10 (0.30) |
| $\sigma$ | 1.60 (0.011) | 1.66 (0.02) | 1.42 (0.015) |

| **Parameters**  **Males** | **Prior 1** | **Prior 2** | **Prior 3** |
| --- | --- | --- | --- |
| β_0_ | 27.25 (0.17) | 25.91 (0.47) | 26.510 (0.17) |
| β_1_ | 0.63 (0.01) | 0.58 (0.03) | 0.62 (0.011) |
| β_2_ | -0.44 (0.05) | -0.45 (0.04) | -0.48 (0.02) |
| CP | 21.30 (0.62) | 22.95 (0.53) | 21.83 (0.46) |
| $\sigma_{\beta0}$ | 2.44 (0.05) | 2.68 (0.05) | 2.38 (0.06) |
| $\sigma_{\beta1}$ | 0.062 (0.005) | 0.054 (0.004) | 0.07 (0.004) |
| $\sigma_{\beta2}$ | 0.08 (0.007) | 0.08 (0.009) | 0.05 (0.01) |
| $\sigma_{CP}$ | 3.34 (1.14) | 4.79 (1.21) | 5.79 (0.21) |
| $\sigma_{\beta1\beta2}$ | 0.017 (0.20) | 0.13 (0.22) | 0.14 (0.25) |
| $\sigma$ | 1.50 (0.013) | 1.36 (0.02) | 1.21 (0.02) |

**Table S2** Posterior estimates of the parameters given in table 1, using the 3 different sets of priors.
