# Supplementary material for: Bayesian hierarchical piecewise regression models: a tool to detect trajectory divergence between groups in long-term observational studies
Source: BMC Med Res Methodol. 2017 Jun 6;17:86. doi: 10.1186/s12874-017-0358-9 (PMC5461770; doi:10.1186/s12874-017-0358-9)
Supplement: Supplementary file 5 — Log-likelihood profiling method and R-code for the choice of priors of the changepoint mean. (μcp). (DOCX 14 kb) [file 12874_2017_358_MOESM5_ESM.docx]

**Additional file 5.**

**Log-likelihood profiling method and R-code for the choice of priors of the changepoint mean (μ_cp)._**

When investigating the effect of prior choice (S4 File) on the posterior parameters estimates when fitting the unconditional BMI trajectory model, the priors considered for the change point mean in the set ‘prior 3’, was based on the estimate of the fixed change points for each sex, based on profile-log likelihood in the maximum likelihood. The procedure passes the breakpoint as a model parameter, and minimizes the deviance of the fitted model conditional upon the breakpoint using the optimize function in R. This maximizes the profile log likelihood for the breakpoint, and the function interior to the wrapper (lmer() in this case) finds the maximum likelihood estimates conditional upon the changepoint, so the whole procedure finds the joint maximum likelihood estimates for all the parameters. The following R code was used for each sex to generate the population-average estimates of the knots in the unconditional BMI trajectory model:

library(lme4)

# NOTE: In this formulation, intercept is expected BMI value at the changepoint itself

#### Estimation of sex-specific average breakpoint based on deviance minimization:

#Basis functions

bp = 4

b1 <- function(x, bp) ifelse(x < bp, bp - x, 0)# before slope

b2 <- function(x, bp) ifelse(x < bp, 0, x - bp) # after slope

# Wrapper for Mixed effects model with variable break point

foo <- function(bp)

{

mod <- lmer(bmi ~ b1(age, bp) + b2(age, bp) + (b1(age, bp) + b2(age, bp) | id), data = T2DMdataF)

deviance(mod)

}

search.range <- c(min(T2DMdataF$age)+0.5,max(T2DMdataF$age)-0.5) # Vector of Possible knots (=the potential ages at which the transition in growth rate occurs)

foo.opt <- optimize(foo, interval = search.range)

bp <- foo.opt$minimum # breakpoint that minimizes deviance

print(bp)#=16.328 year for females (22 years for males)
