# Supplementary material for: Bayesian hierarchical piecewise regression models: a tool to detect trajectory divergence between groups in long-term observational studies
Source: BMC Med Res Methodol. 2017 Jun 6;17:86. doi: 10.1186/s12874-017-0358-9 (PMC5461770; doi:10.1186/s12874-017-0358-9)
Supplement: Supplementary file 6 — Annotated RJAGS sample code to fit a type 1 trajectory divergence model with a fully unstructured 4 by 4 covariance matrix for the random growth parameters. (DOCX 14 kb) [file 12874_2017_358_MOESM6_ESM.docx]

**Additional file 6.**

**Annotated RJAGS sample code to fit a type 1 trajectory divergence model with a fully unstructured 4 by 4 covariance matrix for the random growth parameters.**

Library(rjags)

Library(mvtnorm)

########################################

### Type 1 divergence Bayesian model :

########################################

cat("

data

{

# Zero means for random effect on the 4 growth parameters:

zero.u[1] <- 0 #mean ranef intercept

zero.u[2] <- 0 #mean ranef slope before CP

zero.u[3] <- 0 #mean ranef slope AFTER CP

zero.u[4] <- 0 #mean ranef CP timing

}

model

{

# Random intercept,slopes and CP for each subject:

for( j in 1:M ) #

{

u[j,1:4] ~ dmnorm(zero.u[1:4],invSigma.u[1:4,1:4])# invSigma.u = full 4 x 4 precision matrix for random effects

}

# Define likelihood for each observational unit:

for( k in 1:N )

{

# Linear-linear piecewise model (b2 switches off once b3 switches on) :

mu[k] <- b[1]+u[id[k],1]+(b[2]+u[id[k],2])*Age[k]*(1-step(Age[k]-(CP+betaGroup*(Grp[k]-1))-u[id[k],4]))

+(b[3]+u[id[k],3])*step(Age[k]-CP-betaGroup*(Grp[k]-1)-u[id[k],4])*(Age[k]-CP-betaGroup*(Grp[k]-1)-u[id[k],4])

+b[2]*(CP+betaGroup*(Grp[k]-1)+u[id[k],4])*step(Age[k]-CP-betaGroup*(Grp[k]-1)-u[id[k],4])

y[k] ~ dnorm(mu[k],tau.e)

# Other formulatiOn of Random CP (b2 = common slope, b3 =change to b2 after CP)

# mu[k] <- b[1]+u[id[k],1]+(b[2]+u[id[k],2])*Age[k]+(b[3]+u[id[k],3])*step(Age[k]-CP[Grp[k]]-u[id[k],4])*(Age[k]-CP[Grp[k]]-u[id[k],4])

#y[k] ~ dnorm(mu[k],tau.e)

}

# Fixed intercept, slopes and CP (uninformative)

b[1] ~ dnorm(0.0,1.0E-5)

b[2] ~ dnorm(0.0,1.0E-5)

b[3] ~ dnorm(0.0,1.0E-5)

CP~ dnorm(16.0,1.0E-5)# CP group 1

betaGroup~ dnorm(0.0,1.0E-5) # difference to CP in group2

# Residual variance

tau.e ~dgamma(0.01,0.01)

sigma.e <-1/1/sqrt( tau.e )

# Define 4x4 variance-covariance matrix of subject ranefs

#u[j,1:4] ~ dmnorm(zero.u,invSigma.u)

invSigma.u[1:4,1:4] ~ dwish(R[1:4,1:4],4) # precision matrix

Sigma.u[1:4,1:4]<-inverse(invSigma.u[1:4,1:4])# var-cov matrix

Sigma.u1<-Sigma.u[1,1] # variance intercept

Sigma.u2<-Sigma.u[2,2] # variance first slope

Sigma.u3<-Sigma.u[3,3]# variance second slope

Sigma.u4<-Sigma.u[4,4] ## variance CP

# correlations

r12<-Sigma.u[1,2]/sqrt( Sigma.u1* Sigma.u2)

r13<-Sigma.u[1,3]/sqrt( Sigma.u1* Sigma.u3)

r14<-Sigma.u[1,4]/sqrt( Sigma.u1* Sigma.u4)

r23<-Sigma.u[2,3]/sqrt( Sigma.u2* Sigma.u3)

r24<-Sigma.u[2,4]/sqrt( Sigma.u2* Sigma.u4)

r34<-Sigma.u[3,4]/sqrt( Sigma.u3* Sigma.u4)

}",file="otherCPmodel.jag")

###########################################################

### fit models

###########################################################

track.variables<-c("b","CP" ,"betaGroup","sigma.e","Sigma.u1","Sigma.u2","Sigma.u3","Sigma.u4",

"r12","r13","r14","r23","r24","r34")

head(MCdata)

inits=list(b=c(26,0.5,-0.5),CP=16,betaGroup=0,tau.e=100,invSigma.u=matrix(c(10,0,0,0,0,0.1,0,0,0,0,0.1,0,0,0,0,10),nrow=4))

R<- matrix(c(0.1,0,0,0,0,0.1,0,0,0,0,0.1,0,0,0,0,0.1),nrow=4)

data= list("y" = MCdata$mu,

"Age" = MCdata$age-25,

"id" = as.integer(MCdata$id),

"Grp" = as.integer(MCdata$groupnames),

"N" = as.integer(nrow(MCdata)),

"M" = as.integer(length(unique(MCdata$id))),

"R"=R)

# fit model :

otherCPmodel <- jags.model(

file = "otherCPmodel.jag",

data = data,

inits=inits,

n.chains = 4,

n.adapt = 500)

update(otherCPmodel,1000)

sampleotherCPmodel<- coda.samples(otherCPmodel,

var = track.variables,

n.iter = 1000,

thin = 10 )

summary(sampleotherCPmodel)
