# Supplementary material for: Bayesian hierarchical piecewise regression models: a tool to detect trajectory divergence between groups in long-term observational studies
Source: BMC Med Res Methodol. 2017 Jun 6;17:86. doi: 10.1186/s12874-017-0358-9 (PMC5461770; doi:10.1186/s12874-017-0358-9)
Supplement: Supplementary file 7 — Posterior mean parameter estimates for best fitting birth cohort-adjusted trajectory model for each sex (Model E for females and Model A for males). (DOCX 16 kb) [file 12874_2017_358_MOESM7_ESM.docx]

**Additional file 7**

| **Parameters** | **Females (Model E)** | **Males (Model A)** |
| --- | --- | --- |
| β_0_ | 26.92 (0.27) | 26.510 (0.17) |
| β_1_ | 0.63 (0.017) | 0.62 (0.011) |
| β_2_ | -0.45 (0.02) | -0.48 (0.01) |
| CP | 16.04 (0.5) | 21.83 (0.46) |
| β_2_ yob62 | 0.011 (0.01**) ^ξ^** | - |
| β_2_ yob65 | 0.01 (0.01) **^ξ^** | - |
| β_2_ yob68 | 0.03 (0.025) ^ξ^ | - |
| β_2_ yob74 | **0.06 (0.02)** | - |
| β_2_ yob77 | 0.01 (0.03) ^ξ^ | - |
| CP yob62 | 0.24 (0.69) ^ξ^ | - |
| CP yob65 | 0.53 (0.4) ^ξ^ | - |
| CP yob68 | **2.89** **(0.8)** | - |
| CP yob74 | 0.33 (0.8) ^ξ^ | - |
| CP yob77 | 0.7 (0.73) ^ξ^ | - |
| $\sigma_{\beta0}$ | 2.08 (0.05) | 2.38 (0.06) |
| $\sigma_{\beta1}$ | 0.01 (0.003) | 0.07 (0.004) |
| $\sigma_{\beta2}$ | 0.18 (0.005) | 0.05 (0.01) |
| $\sigma_{CP}$ | 3.22 (1.07) | 5.79 (0.21) |
| $\sigma_{\beta1\beta2}$ | 0.11 (0.05) | 0.14 (0.03) |
| $\sigma$ | 1.37 (0.03) | 1.21 (0.02) |
| $\beta_{log(insulin)}$ | 1.00 (0.04) | 0.99 (0.03) |

Posterior mean parameter estimates for best fitting birth cohort-adjusted trajectory model for each sex; Model E for females and Model A for males. Posterior standard deviations in brackets. **^ξ^** 95% credible interval cover zero (reference cohort is 1971). Reported β_0_  coefficients are in kg/m^2^. All β_1_ and β_2_ coefficients are in kg/m^2^  per year, CP coefficients are in years. σ coefficients are standard deviations for the corresponding growth parameters and the residual error. $\beta_{log(insulin)}$ coefficients are in kg/m^2^ for a 1 sd increase in log(insulin) level.

***Note***: Because inter-cohort differences in BMI development over age were not overly pronounced, we did not adjust for the cohort effect when estimating the effect of “T2DM status” on BMI.
