# Supplementary material for: Bayesian hierarchical piecewise regression models: a tool to detect trajectory divergence between groups in long-term observational studies
Source: BMC Med Res Methodol. 2017 Jun 6;17:86. doi: 10.1186/s12874-017-0358-9 (PMC5461770; doi:10.1186/s12874-017-0358-9)
Supplement: Supplementary file 8 — Results of mixed models with age as a categorical predictor and log-insulin as a continuous predictor: LS means contrasts (No adult T2DM vs. adult T2DM) and significance at each age averaged over- (Table S1.) or adjusted for the levels of sex (Table S2.) and pairwise comparisons of Least-square means of BMI and 95% CIs at each age in each T2DM status group averaged over levels of sex (Figure S1.) at each age in each T2DM status group and sex group combination (M = males, F-females, 1 = No adult T2DM, 2 = adult T2DM) (Figure S2.) and adjusted for log(insulin). (DOCX 549 kb) [file 12874_2017_358_MOESM8_ESM.docx]

**Additional file 8**.

**Table S1**. Results of mixed models with age as a categorical predictor and log-insulin as a continuous predictor: LS means contrasts (No adult T2DM vs. adult T2DM) and significance at each age, averaged over levels of sex.

| **Age** | **Estimate*** (kg/m^2^) | **SE** | **t-ratio** | **p-value** |
| --- | --- | --- | --- | --- |
| 3 | 1.52 | 1.04 | 1.455 | 0.14 |
| 6 | 0.54 | 0.71 | 0.76 | 0.44 |
| 9 | 0.26 | 0.56 | 0.47 | 0.63 |
| 12 | -0.87 | 0.50 | -1.72 | 0.0851 |
| 15 | -1.11 | 0.44 | -2.52 | **0.0115** |
| 18 | -1.30 | 0.42 | -3.05 | **0.002** |
| 21 | -1.45 | 0.51 | -2.8 | **0.005** |
| 24 | -1.56 | 0.59 | -2.64 | **0.008** |
| 27 | -0.93 | 1.33 | -0.70 | 0.48 |
| 30 | -4.01 | 0.72 | -5.54 | **<.0001** |
| 33 | -2.62 | 0.55 | -4.77 | **<.0001** |
| 37 | -5.00 | 0.51 | -9.73 | **<.0001** |
| 40 | -4.39 | 0.43 | -10.17 | **<.0001** |
| 43 | -3.19 | 0.49 | -6.4 | **<.0001** |
| 46 | -3.39 | 0.47 | -7.17 | **<.0001** |
| 49 | -4.94 | 0.56 | -8.81 | **<.0001** |

*: the estimated contrast is obtained from a log(insulin) adjusted model (Degree of freedom is equal to 2339)

**Figure S1.** Pairwise comparisons of Least-square means of BMI and 95% CIs at each age in each T2DM status group, averaged over levels of sex


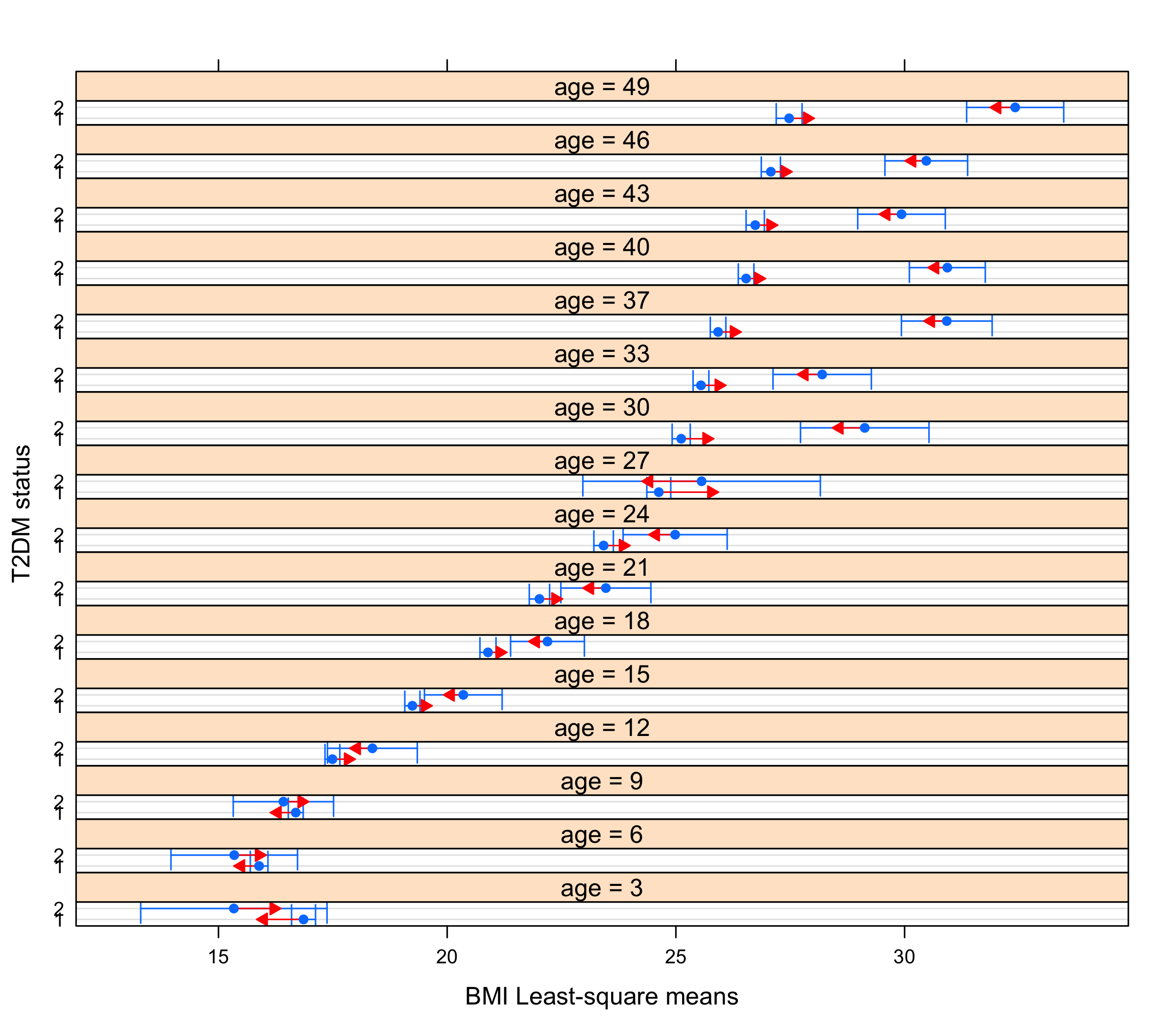


Blue intervals represent the 95% CI for each predicted BMI LS-mean. Red comparisons arrows show which LS-means differ significantly from one another based on whether or not they overlap. Comparisons are made using a 0.05 Tukey-adjusted significance level.

**Table S2.** LS means contrasts (No adult T2DM vs. adult T2DM) and significance at each age, at each level of sex.

| **Age** | **Estimate**  (kg/m^2^) | **SE** | **t-ratio** | **p-value** |
| --- | --- | --- | --- | --- |
| ***Females*** | | | | |
| 3 | 0.76 | 1.67 | 0.456 | 0.64 |
| 6 | -1.01 | 1.01 | -0.10 | 0.91 |
| 9 | -0.28 | 0.74 | -0.37 | 0.70 |
| 12 | -0.53 | 0.64 | -0.87 | 0.41 |
| 15 | -0.6 | 0.58 | -1.07 | 0.28 |
| 18 | -1.29 | 0.61 | -2.1 | **0.03** |
| 21 | -1.61 | 0.74 | -2.2 | **0.003** |
| 24 | -2.48 | 0.85 | -2.9 | **0.003** |
| 27 | -0.18 | 1.37 | -0.13 | 0.89 |
| 30 | -4.01 | 0.94 | -5.54 | **<.0001** |
| 33 | -2.62 | 0.55 | -5.1 | **<.0001** |
| 37 | -5.45 | 0.78 | -8.1 | **<.0001** |
| 40 | -5.93 | 0.60 | -9.8 | **<.0001** |
| 43 | -3.69 | 0.71 | -5.15 | **<.0001** |
| 46 | -5.18 | 0.4 | -7.35 | **<.0001** |
| 49 | -6.4 | 0.81 | -7.87 | **<.0001** |
| ***Males*** | | | | |
| 3 | 2.28 | 1.35 | 1.82 | 0.06 |
| 6 | 1.19 | 0.99 | 1.20 | 0.22 |
| 9 | 0.82 | 0.84 | 0.97 | 0.33 |
| 12 | -1.21 | 0.65 | -1.50 | 0.12 |
| 15 | -1.60 | 0.44 | -2.43 | **0.015** |
| 18 | -1.31 | 0.57 | -2.27 | **0.03** |
| 21 | -1.28 | 0.71 | -1.8 | 0.06 |
| 24 | -0.64 | 0.81 | -0.78 | 0.43 |
| 27 | -1.68 | 2.23 | -0.74 | 0.47 |
| 30 | -2.81 | 1.09 | -2.51 | **0.009** |
| 33 | -1.26 | 0.78 | -1.6 | 0.1097 |
| 37 | -4.50 | 0.41 | -5.83 | **<.0001** |
| 40 | -2.9 | 0.68 | -4.6 | **<.0001** |
| 43 | -3.19 | 0.69 | -3.8 | **<.0001** |
| 46 | -3.39 | 0.62 | -2.5 | **0.01** |
| 49 | -3.47 | 0.76 | -4.51 | **<.0001** |

**Figure S2.** Pairwise comparisons of Least-square means of BMI and 95% CIs at each age in each T2DM status group and sex group combination


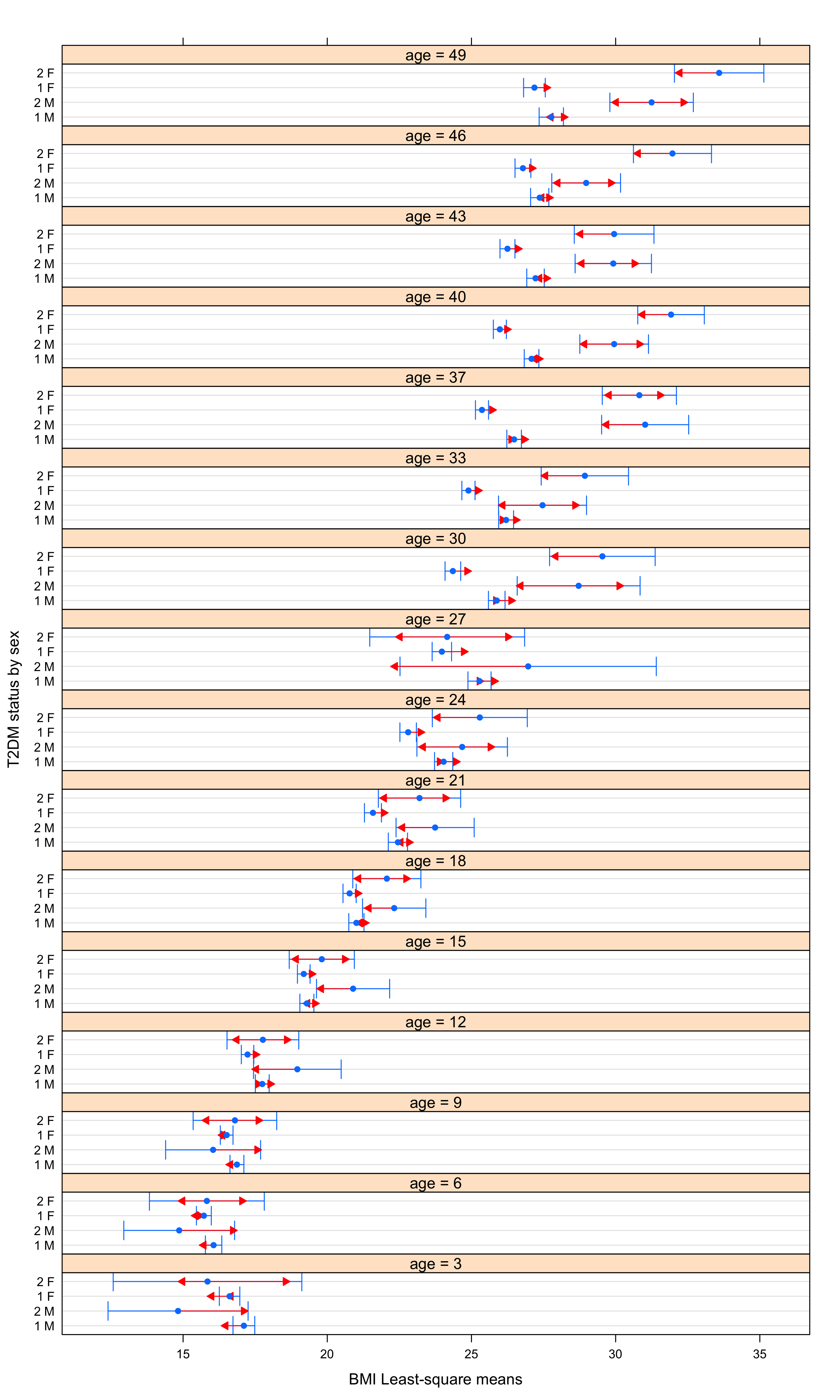


Blue intervals represent the 95% CI for each predicted BMI LS-mean. Red comparisons arrows show which LS-means differ significantly from one another based on whether or not they overlap. Comparisons are made using a 0,05 Tukey-adjusted significance level.
